# Long-Term Kidney Outcomes After SARS-CoV-2 Infection in Children Aged 0–12 Years: A Systematic Review

**DOI:** 10.3390/children13010075

**Published:** 2026-01-02

**Authors:** Saad Alhumaid, Abdullah Abdulrahman Alkhamees, Nourah Al Dossary, Anwar A. Almuslim, Rabab Abbas Majzoub, Qasem M. Alalwan, Mohammed Jassim Alsaeed, Fahad Mohammed Aljowaisem, Manahi Ayadh Alqahtani, Abdulmohsen Ibrahim Alamer, Muath Ibrahim ALDuhailan, Dawood Adnan Al Nasser, Mohammed S. Almuhanna, Mustafa A. Al-Kamees, Hassan Ali Alhadab, Ali Ahmed Alsultan, Ali N. Bukhamseen, Abdulaziz Abdullah Alabdullah, Kawther S. Alhaddad, Murtadha A. Alhumaid, Hassan M. Almusabeh, Yasin S. Almubarak, Rugayah Ahmed AlShayeb, Dalal Ahmed Alnami, Yaqoub Yousef Alatiyyah, Zainab Al Alawi, Muneera Alabdulqader

**Affiliations:** 1 School of Pharmacy, University of Tasmania, Hobart 7000, Australia; 2 Pharmaceutical Care Department, King Abdulaziz Hospital, Ministry of National Guard Health Affairs, Al-Ahsa 36428, Saudi Arabia; alkhameesab@mngha.med.sa (A.A.A.); alsaeedmo3@mngha.med.sa (M.J.A.); aljowesimfa@mngha.med.sa (F.M.A.); alqahtanima21@mngha.med.sa (M.A.A.); alamerab8@mngha.med.sa (A.I.A.); alduhailanmu@mngha.med.sa (M.I.A.); 3 General Surgery Department, King Fahad Hofuf Hospital, Al-Ahsa Health Cluster, Ministry of Health, Al-Ahsa 36441, Saudi Arabia; nofaldossary@moh.gov.sa; 4 Pharmacy Department, Maternity and Children Hospital, Ministry of Health, Al-Ahsa 36422, Saudi Arabia; aaalmuslim@moh.gov.sa (A.A.A.); msalmuhanna@moh.gov.sa (M.S.A.); abokhamsin@moh.gov.sa (A.N.B.); abalabdullah@moh.gov.sa (A.A.A.); 5 Department of Pediatrics, College of Medicine, King Faisal University, Al-Ahsa 31982, Saudi Arabia; rmajzoub@kfu.edu.sa; 6 Radiology Department, King Fahad Hofuf Hospital, Al-Ahsa Health Cluster, Ministry of Health, Al-Ahsa 36441, Saudi Arabia; qalalwan@moh.gov.sa; 7 Long-Term Care Department, Oyun City General Hospital, Al-Ahsa Health Cluster, Ministry of Health, Al-Ahsa 36312, Saudi Arabia; dalnasser@moh.gov.sa; 8 Hazm Al-Mubarraz Health Center, Al-Ahsa Health Cluster, Ministry of Health, Al-Ahsa 36341, Saudi Arabia; mualkhamis@moh.gov.sa; 9 Ambulatory Transportation Administration, Al-Ahsa Health Cluster, Ministry of Health, Al-Ahsa 36421, Saudi Arabia; hassan-1436hs@moh.gov.sa; 10 Medical Supply Store, Aloyoon General Hospital, Ministry of Health, Al-Ahsa 36312, Saudi Arabia; aaalsultan@moh.gov.sa; 11 Pediatric Nursing Ward, Maternity and Children Hospital, Al-Ahsa Health Cluster, Ministry of Health, Al-Ahsa 36422, Saudi Arabia; ksalhaddad@moh.gov.sa; 12 Pharmacy Department, Aloyoon General Hospital, Ministry of Health, Al-Ahsa 36312, Saudi Arabia; mualhumaid@moh.gov.sa; 13 Administration of Pharmaceutical Care, Al-Ahsa Health Cluster, Ministry of Health, Al-Ahsa 36421, Saudi Arabia; halmusabeh@moh.gov.sa; 14 Regional Medical Supply, Al-Ahsa Health Cluster, Ministry of Health, Al-Ahsa 36361, Saudi Arabia; yalmubarak@moh.gov.sa; 15 Pharmacy Department, King Fahad Hofuf Hospital, Al-Ahsa Health Cluster, Ministry of Health, Al-Ahsa 36441, Saudi Arabia; ralshayeb@moh.gov.sa (R.A.A.); dalnami@moh.gov.sa (D.A.A.); 16 Department of Pharmacy, Hereditary Blood Diseases Centre, Ministry of Health, Al-Ahsa 36422, Saudi Arabia; yalatiyyah@moh.gov.sa; 17 Division of Allergy and Immunology, College of Medicine, King Faisal University, Al-Ahsa 31982, Saudi Arabia; zalalwi@kfu.edu.sa; 18 Pediatric Nephrology Specialty, Pediatric Department, Medical College, King Faisal University, Al-Ahsa 31982, Saudi Arabia; malabdulqader@kfu.edu.sa

**Keywords:** children, COVID-19, kidney, long-term, outcomes, renal, SARS-CoV-2, systematic review

## Abstract

**Highlights:**

**What are the main findings?**

- Evidence on long-term kidney outcomes after SARS-CoV-2 infection in children aged 0–12 years is scarce, with only a small number of studies providing extractable age-specific data.
- Available MIS-C and mild COVID-19 cohorts generally show favourable short- to medium-term renal recovery, but larger EHR studies suggest potential long-term kidney risk in broader paediatric populations without age-stratified estimates for young children.

**What is the implication of the main finding?**

- Current evidence is insufficient to draw firm conclusions about long-term kidney outcomes in children aged 0–12 years, highlighting the need for age-stratified prospective studies with extended follow-up.
- Structured renal follow-up may be warranted for children with severe COVID-19, MIS-C, or acute kidney injury until more definitive long-term data become available.

**Abstract:**

**Background:** Acute kidney injury (AKI) is increasingly recognised in children with acute COVID-19 and multisystem inflammatory syndrome in children (MIS-C), yet the long-term renal consequences in younger paediatric populations remain unclear. Most studies focus on acute illness or mixed-age cohorts, with limited data specific to children aged 0–12 years. **Objectives:** This study aimed to systematically identify, evaluate, and synthesise evidence on post-acute (≥30 days) and long-term (≥90 days) kidney outcomes following SARS-CoV-2 infection or MIS-C in children aged 0–12 years, including chronic kidney disease (CKD), eGFR decline, proteinuria, haematuria, hypertension, and need for kidney replacement therapy. **Methods:** We searched MEDLINE, Embase, CINAHL, and PubMed (December 2019–30 November 2025), following PRISMA 2020 guidelines and a registered PROSPERO protocol (CRD420251241949). Observational studies reporting kidney outcomes ≥30 days post-infection in children aged 0–12 years were included. Risk of bias was assessed using the Newcastle–Ottawa Scale or ROBINS-I. Owing to heterogeneity and absence of ≥3 comparable datasets, a narrative synthesis was performed. **Results:** Seven studies met inclusion criteria (five MIS-C cohorts, two acute COVID-19 cohorts). Only a subset provided extractable data specific to children aged 0–12 years. Follow-up ranged from 30 days to 12 months; four studies reported outcomes ≥ 180 days. Across all studies, no incident CKD, sustained eGFR decline, or kidney replacement therapy were reported among children completing long-term follow-up; however, most long-term outcome data were derived from MIS-C cohorts with median ages around 8–11 years that included some adolescents, rather than exclusively children aged 0–12 years. One MIS-C study reported long-term hypertension in 14% of children. A cross-sectional Italian cohort of mild COVID-19 demonstrated hyperfiltration, proteinuria, and microhaematuria at ~3 months, though chronicity could not be assessed due to absence of baseline values. A large US EHR-based cohort identified increased CKD risk after COVID-19 in the broader < 21-year population; however, 0–12-year-specific event counts were not reported, preventing quantitative synthesis for young children. **Conclusions:** Evidence on long-term kidney outcomes after SARS-CoV-2 infection in children aged 0–12 years remains limited, and only a small subset of studies provided extractable, age-specific data. On the other hand, MIS-C cohorts generally show favourable renal recovery, small sample sizes, lack of control groups, and short follow-up restrict confidence in these findings. Large paediatric EHR studies suggest potential long-term renal risk in broader paediatric populations, highlighting the need for age-stratified, prospective cohorts with serial eGFR, urine studies, and blood pressure assessments. Until definitive evidence emerges, structured renal follow-up may be warranted for children with AKI or MIS-C during COVID-19.

## 1. Introduction

Coronavirus disease 2019 (COVID-19), caused by severe acute respiratory syndrome coronavirus 2 (SARS-CoV-2), has affected children worldwide since late 2019 [1]. Although paediatric infections are often mild, a subset of children develop significant complications involving the respiratory, cardiovascular, and renal systems [2,3]. A post-infectious hyperinflammatory condition known as multisystem inflammatory syndrome in children (MIS-C), also referred to as paediatric inflammatory multisystem syndrome temporally associated with SARS-CoV-2 (PIMS-TS) [4], was recognised in 2020 and is characterised by persistent fever, elevated inflammatory markers, multiorgan involvement, and recent SARS-CoV-2 exposure [5,6]. MIS-C commonly affects school-aged children and may present with acute kidney injury (AKI), electrolyte disturbances, hypotension, and shock requiring intensive care [7,8].

Kidney involvement has been increasingly reported in both acute COVID-19 and MIS-C in paediatric populations, with manifestations ranging from transient creatinine elevation to KDIGO-defined acute kidney injury [9]. Children aged 0–12 years differ from adolescents in renal maturation, blood pressure physiology, comorbidity profiles, and pubertal or hormonal development, all of which may influence susceptibility to kidney injury and recovery trajectories [10,11,12]. Proposed mechanisms include cytokine-mediated tubular injury, endothelial dysfunction, hypovolaemia, and systemic inflammation, consistent with established AKI pathophysiology [13]. International bodies such as the World Health Organization (WHO) and KDIGO emphasise the need to monitor post-acute and chronic sequelae of COVID-19, particularly as organ involvement may persist beyond 12 weeks [14,15]. However, long-term renal outcomes in children, especially those aged 0–12 years, remain poorly defined.

Notably, much of the existing paediatric literature on COVID-19-related kidney outcomes reports mixed age cohorts, often combining infants, children, and adolescents, which limits the clinical interpretability of long-term renal outcomes specifically for younger children. Most available studies focus on acute illness, lack age-stratified analyses, or do not extend follow-up beyond 90 days. Given the developmental and physiological differences between younger children and adolescents, extrapolation of long-term renal risk across paediatric age groups may be inappropriate [10,11,12].

Systematic reviews conducted according to the Preferred Reporting Items for Systematic Reviews and Meta-Analyses (PRISMA) 2020 and guided by the PICOS framework provide a structured approach for synthesising heterogeneous evidence and ensuring transparency in study selection [16,17]. The use of established definitions from WHO [15,18], the Centers for Disease Control and Prevention (CDC) [5], the Royal College of Paediatrics and Child Health (RCPCH) [19], and KDIGO guidelines for CKD and AKI [20,21] further supports consistency in outcome interpretation.

As a result, whether children experience chronic kidney disease (CKD), persistent proteinuria or haematuria, hypertension, estimated glomerular filtration rate (eGFR) decline, or dialysis dependence after SARS-CoV-2 infection remains unclear. Restricting the analysis to a biologically and developmentally more homogeneous age group reduces confounding related to pubertal development and comorbidity burden, thereby strengthening the clinical relevance of age-specific kidney outcome assessment. This systematic review therefore aims to identify and critically evaluate long-term kidney outcomes following SARS-CoV-2 infection or MIS-C specifically in children aged 0–12 years.

## 2. Methods

### 2.1. Design

This systematic review was conducted in accordance with PRISMA 2020 guidelines [17] and was prospectively registered with PROSPERO (CRD420251241949). Eligibility criteria were defined using the PICOS framework to ensure a focused and transparent approach [16]. A summary of PICOS components is provided in Table 1.

The PRISMA 2020 checklist was used (see Supplementary Materials) [17].

### 2.2. Data Sources and Search Strategy

A comprehensive search of MEDLINE, Embase, CINAHL, and PubMed was performed for studies published from December 2019 to 30 November 2025. The strategy combined controlled vocabulary and free-text terms relating to SARS-CoV-2, COVID-19, kidney or renal outcomes, and children. Full database-specific search syntax is provided in Supplementary Table S1.

Search results were imported into EndNote 21.2 for de-duplication and uploaded to Covidence for screening. Reference lists of included studies and relevant systematic reviews were hand-searched, and backward/forward citation tracking was performed. Preprints were screened for emerging evidence, but only peer-reviewed full-text articles were included. Grey literature, conference abstracts, and non-English publications were excluded.

### 2.3. Eligibility Criteria

#### 2.3.1. Inclusion Criteria

Studies were eligible if they:

- Included children aged 0–12 years with confirmed or probable SARS-CoV-2 infection (acute COVID-19 or MIS-C/PIMS-TS);
- Reported kidney outcomes assessed at post-acute (≥30 days) or long-term (≥90 days) time points after infection, including single post-acute cross-sectional assessments;
- Used observational designs with longitudinal follow-up or post-acute cross-sectional evaluation (prospective or retrospective cohort, case–control, registry/EHR cohort);
- Provided extractable data for children ≤ 12 years or had a median/mean cohort age ≤ 12 years.

#### 2.3.2. Exclusion Criteria

We excluded:

- Randomised trials, case reports, case series with <7 participants, cross-sectional studies without follow-up, qualitative studies, reviews, editorials, commentaries;
- Purely acute cross-sectional studies reporting kidney outcomes only during the initial infection period (<30 days);
- Studies without identifiable SARS-CoV-2 infection status;
- Non-peer-reviewed manuscripts and non-English publications.

### 2.4. Definitions

Operational definitions were applied for consistency across studies:

- SARS-CoV-2 infection: confirmed by PCR, antigen test, serology, or clinical diagnosis according to national/WHO criteria [15].
- MIS-C/PIMS-TS: defined using WHO, CDC, or RCPCH criteria [5,6,19].
- Age definition (0–12 years): children were defined as those aged 0–12 years to capture a biologically homogeneous paediatric group with shared renal and immune developmental profiles [1,22]. Adolescents were excluded because their near-adult kidney maturation, blood pressure physiology, and inflammatory responses differ substantially from younger children, which could confound age-specific renal outcome assessment [11,12,23].
- Long-term follow-up: ≥90 days after infection or hospital discharge, in line with WHO post-COVID-19 condition definitions [18].
- CKD: KDIGO 2012 criteria (eGFR < 90 mL/min/1.73 m^2^ or markers of kidney damage for ≥3 months) [21].
- AKI: KDIGO or AKIN creatinine-based definitions when reported [20].
- Proteinuria/haematuria: assessed per individual study methods (dipstick, ACR, PCR, microscopy) [21].
- Hypertension: defined according to paediatric blood pressure percentiles or thresholds specified in each study [10].

Because definitions and measurement methods for kidney outcomes varied across included studies, outcome definitions were not statistically harmonised. Instead, outcomes were interpreted using established reference standards where available (KDIGO criteria for CKD and AKI) and study-specific definitions for proteinuria, haematuria, and hypertension. A consolidated description of diagnostic criteria and measurement methods is presented in Supplementary Table S2.

### 2.5. Data Extraction

Data extraction was performed using a standardised template tailored for this review. Extracted variables included study characteristics (author, year, country, design, setting), participant demographics (age distribution, sex, comorbidities, pre-existing kidney disease), SARS-CoV-2 phenotype (acute COVID-19 or MIS-C/PIMS-TS), AKI occurrence during the acute phase, and follow-up duration categorised as 30–89 days (post-acute), 90–179 days (early long-term), or ≥180 days (long-term). Kidney outcomes were collected at the longest available follow-up and included eGFR values, CKD incidence or progression, proteinuria, haematuria, hypertension, and kidney replacement therapy. When studies reported multiple follow-up timepoints or intermediate measurements, these were recorded in Supplementary Extraction Files.

Four reviewers independently extracted all data. Any disagreements during study selection or data extraction were resolved through discussion and consensus among the reviewers; when consensus could not be reached, a senior reviewer adjudicated. When multiple reports originated from the same cohort, data were consolidated into a single entry. Where measures of variance (e.g., standard deviations or confidence intervals) were missing, they were calculated from available information or obtained from study authors when possible.

### 2.6. Risk of Bias Assessment

Risk of bias in the included studies was assessed independently by two reviewers using the Newcastle–Ottawa Scale (NOS) for cohort and case–control studies, as recommended for evaluating the quality of non-randomised studies in systematic reviews [24]. For more complex observational designs, such as quasi-experimental or multi-level registry analyses, risk of bias was assessed using the Risk Of Bias In Non-randomised Studies of Interventions (ROBINS-I) tool, which evaluates bias across seven domains including confounding, participant selection, classification of interventions, deviations from intended exposure, missing data, outcome measurement, and selection of reported results [25]. Domains evaluated for all studies included participant selection, comparability of groups, outcome measurement, and adequacy of follow-up. Any disagreements between reviewers were resolved through discussion and consensus; when consensus could not be reached, a senior reviewer adjudicated (see Supplementary Tables S3 and S4 for the quality assessment of included studies). Studies with complex, large-scale EHR-based designs (e.g., Li et al. [26]) were evaluated using ROBINS-I only; NOS assessment was not applied to these studies.

### 2.7. Data Synthesis

Because no kidney outcome was reported by three or more comparable studies with consistent definitions, follow-up windows, and extractable data for children aged 0–12 years, a meta-analysis was not feasible. Given heterogeneity in outcome definitions, follow-up windows, and the limited availability of age-specific data, formal harmonisation of outcome definitions was also not feasible; therefore, findings were synthesised narratively with explicit acknowledgment of definitional variability across studies. Accordingly, a structured narrative synthesis was conducted in accordance with PRISMA 2020 guidance [17]. Post-acute cross-sectional studies with outcome assessment at ≥30 days were synthesised narratively alongside longitudinal cohorts, while acute-only cross-sectional studies were excluded.

The narrative synthesis involved study-by-study summaries describing design, population characteristics, SARS-CoV-2 phenotype, renal assessments, and follow-up duration. Long-term kidney outcomes were then organised thematically, including CKD incidence, eGFR or creatinine changes, persistent proteinuria or haematuria, hypertension, dialysis or kidney replacement therapy, and composite kidney outcomes. Where applicable, findings were interpreted comparatively between MIS-C and acute COVID-19. The synthesis also considered the consistency of results across studies, noting the direction and magnitude of findings. Sensitivity considerations included heterogeneity in outcome definitions, the inability to isolate results for children aged ≤ 12 years in some datasets, small sample sizes, and the absence of control groups in most MIS-C cohorts.

All analyses were descriptive; no pooled effect estimates, heterogeneity statistics, or publication bias assessments were performed. The graphical abstract was created using https://www.biorender.com/ (accessed on 25 December 2025) (agreement no. DE295QVICG).

## 3. Results

Seven observational studies met the inclusion criteria, comprising five MIS-C cohorts and two acute COVID-19 cohorts, with sample sizes ranging from small single-centre studies to large electronic health record-based analyses [4,26,27,28,29,30,31]. Follow-up durations ranged from 30 days to 12 months. Across studies providing long-term follow-up, no incident chronic kidney disease, no sustained decline in estimated glomerular filtration rate, and no requirement for kidney replacement therapy were reported among children completing follow-up. Data on other long-term renal outcomes were limited, with isolated reports of post-discharge hypertension in MIS-C cohorts and inconsistent findings for proteinuria and haematuria, often derived from mixed paediatric age groups [4,27,28,29,30,31]. The detailed study selection process and individual study results are presented below.

**Figure 1:**
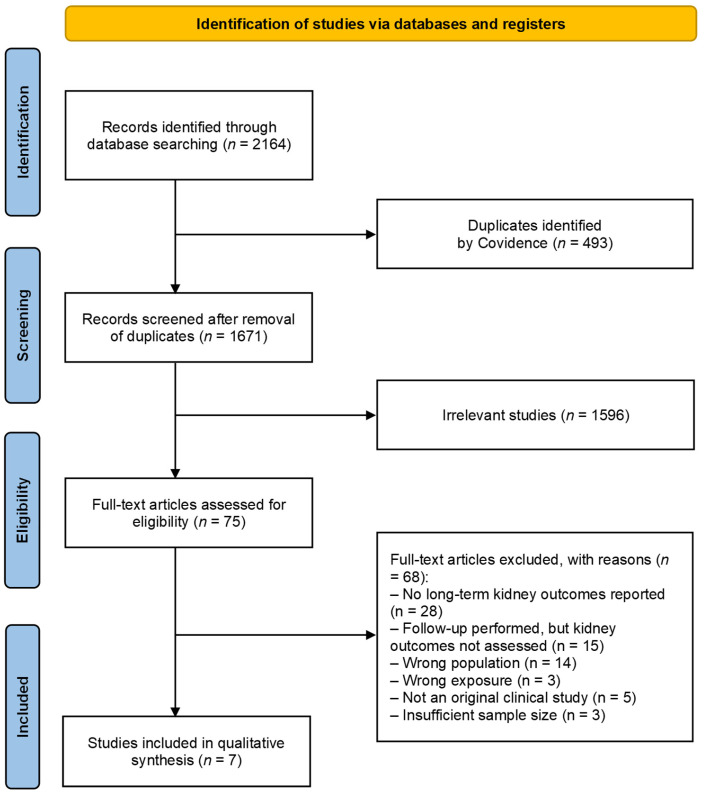
Flow diagram of studies included in the systematic review.

The initial search identified 2164 potentially relevant records. After removal of duplicates, 1671 titles and abstracts were screened. Of these, 1596 records were excluded as clearly irrelevant. The full texts of 75 articles were retrieved and assessed for eligibility. Following detailed review, 68 articles were excluded for not meeting the inclusion criteria (e.g., absence of long-term kidney outcomes, acute-only renal data, wrong population, wrong exposure, or non-original study design). The detailed reasons for exclusion are provided in Supplementary Table S5. Ultimately, seven observational studies met all eligibility criteria and were included in the final systematic review [4,26,27,28,29,30,31]. The study selection process is summarised in Figure 1. The characteristics of the seven included studies are summarised in Table 2. Baseline characteristics of children aged 0–12 years included across all studies are provided in Supplementary Table S6.

The seven included studies comprised retrospective (*n* = 5) [4,26,27,28,29], prospective (*n* = 1) [31], and cross-sectional (*n* = 1) [28] observational designs, conducted across the United States [26,27], Italy [28,29,31], United Kingdom [4], and India [30]. Across these studies, sample sizes ranged from 7 to 487,378 children; however, only a subset provided extractable data specific to children aged 0–12 years [4,30,31]. Most MIS-C cohorts had median ages around 8–11 years but included adolescents up to 17–18 years, and age-stratified renal outcomes for children ≤ 12 years were frequently not separable [4,27,29,30,31]. Five studies evaluated children with MIS-C/PIMS-TS [4,27,29,30,31], while two studies assessed children with acute COVID-19 without MIS-C [26,28]. Only one large EHR-based study included a contemporaneous uninfected control group [26]; all other studies were single-arm cohorts without comparators [4,27,28,29,30,31].

Follow-up duration varied from 30 days to 12 months, with four studies reporting outcomes ≥ 180 days [4,29,30,31]. Kidney outcomes were assessed using combinations of serum creatinine [4,26,27,30,31], creatinine-based AKI definitions (KDIGO or Acute Kidney Injury Network, AKIN) [27,29,30], urine studies (proteinuria or haematuria) [4,28,29,30], blood pressure monitoring [4,27], tubular function markers [29], and, less frequently, creatinine clearance or eGFR [27,28].

Unless otherwise stated, kidney outcomes summarised below reflect results from mixed paediatric cohorts in which children aged 0–12 years constituted the majority, but age-specific outcome data were not always separable. Long-term kidney outcomes for children aged 0–12 years are shown in Table 3. Across all included studies, no incident CKD was reported among children with MIS-C or acute COVID-19 who completed long-term follow-up. No study provided extractable eGFR trajectories for children aged 0–12 years. The only study reporting CKD outcomes did so at the <21-year population level [26]; age-specific values for 0–12 years were not available, and therefore these outcomes were excluded from quantitative synthesis. Accordingly, these findings reflect kidney risk in a broader paediatric and adolescent population (<21 years) and cannot be directly extrapolated to children aged 0–12 years.

Two MIS-C studies, drawn from mixed paediatric cohorts with median ages around 8–11 years that included some adolescents, reported complete resolution of proteinuria and haematuria by 6–12 months [29,30]. One cross-sectional COVID-19 study reported proteinuria and microhaematuria at ~3 months post-infection, but without baseline measurements or repeat follow-up, persistence could not be determined [28].

Only one study reported long-term hypertension: 14% (7/50) of children with MIS-C had elevated blood pressure > 30 days after discharge, with AKI during the acute phase associated with higher odds of post-discharge hypertension [27]. In all other MIS-C cohorts, hypertension was absent or limited to a single child with complex comorbidities [29,30].

No study reported new initiation of dialysis or kidney transplantation during follow-up. All children achieved recovery without requiring KRT.

One MIS-C study systematically evaluated tubular dysfunction (uNAG, TRP, hypophosphatemia, polyuria) [29]. Although TD was common during acute MIS-C, all tubular abnormalities resolved by 6 months, except for mild residual hypertension in one child [29].

Comparative effect estimates from the only study with a control group are presented in Table 4. Only one study included a non-infected control group and reported hazard ratios for kidney outcomes [26]; however, all effect estimates were available only for the full <21-year cohort, not for children aged 0–12 years. Therefore, no valid comparative effect sizes could be extracted for this age group. All other included studies were single-arm cohorts without controls, precluding between-group comparisons.

Meta-analysis was not possible for any kidney outcome. Across the seven included studies [4,26,27,28,29,30,31], no outcome was reported by at least three comparable studies with consistent definitions, follow-up windows, and extractable 0–12-year-specific data. As a result, findings were synthesised narratively in accordance with PRISMA 2020 guidelines [17].

## 4. Discussion

Our systematic review summarises current data on post-acute and long-term kidney outcomes following SARS-CoV-2 infection or MIS-C in paediatric populations. Importantly, although this review focuses on children aged 0–12 years, much of the available long-term evidence derives from MIS-C cohorts with median ages around 8–11 years that included adolescents, limiting the availability of strictly age-specific outcome data. Until very recently, most evidence was limited to case series or small cohorts focusing on AKI in hospitalised children [32]. However, emerging longitudinal data, including large HER-based analyses, are now highlighting potential long-term risks [26].

The most consequential recent evidence comes from a large electronic health record-based cohort including over 1.9 million paediatric patients, which reported an increased risk of new-onset chronic kidney disease (stage ≥ 2: hazard ratio [HR] 1.17; stage ≥ 3: HR 1.35) and sustained eGFR decline following SARS-CoV-2 infection compared with non-infected controls [26]. However, this study did not provide age-stratified estimates specifically for children aged 0–12 years, and all effect estimates were derived from a broader paediatric and adolescent population (<21 years) [26]. Accordingly, while these findings raise important concerns regarding potential long-term renal risk after COVID-19, they cannot be directly extrapolated to younger children. These limitations underscore the need for age-stratified, prospective studies to better characterise long-term kidney outcomes in early childhood.

Complementing this, a single-centre Italian study found that at a median of approximately 3 months post-infection, a surprisingly high proportion of “paucisymptomatic” children showed hyperfiltration, proteinuria, and microhaematuria in a cohort without prior kidney disease [28]. These data raise the possibility of subclinical tubular or glomerular injury that may not immediately manifest as reduced eGFR but could portend future renal stress, especially under additional insults.

It is well established that children recovering from AKI, irrespective of COVID-19, remain at elevated long-term risk of CKD, hypertension, further AKI episodes, and even KRT [33]. This underlines a biological plausibility that even transient AKI during COVID-19 or MIS-C might predispose to chronic sequelae, especially if renal recovery is incomplete or if there is repeated insult (for example recurrent infections, dehydration, or drug exposures).

Furthermore, mechanistic considerations support kidney vulnerability: SARS-CoV-2-associated kidney injury may involve direct infection of proximal tubular epithelial cells (which express viral entry receptors), endothelial injury, complement activation, coagulation abnormalities, inflammatory cytokine storm, and haemodynamic instability, all of which can contribute to acute tubular injury, glomerular damage, or both [34].

MIS-C cohorts generally show favourable renal recovery; however, these findings are based largely on mixed paediatric cohorts with median ages around 10 years and limited age-stratified outcome reporting for children aged 0–12 years. Taken together, these emerging data challenge the previously optimistic notion that children invariably recover without long-term renal sequelae after SARS-CoV-2 infection [3,35]. The large EHR-based study suggests a modest but statistically significant increased risk of CKD, especially in those with preexisting kidney vulnerability or acute kidney injury [26]. However, it is important to note that these risk estimates derive from a combined paediatric and adolescent cohort (<21 years), and age-specific hazard ratios for children aged 0–12 years were not reported, limiting direct inference for this younger age group. The Italian hyperfiltration/proteinuria data imply that subclinical renal injury may be common even after mild infections, and may go unnoticed unless actively sought [28].

However, several caveats remain. First, while the EHR study is large and powerful, follow-up was limited (post-acute up to 365 days), so longer-term trajectories (e.g., over several years) remain unknown [26]. Second, many paediatric AKI series (particularly those from MIS-C) remain small, often lacking long-term follow-up or control groups [36]. Third, the hyperfiltration/proteinuria study had a relatively short median follow-up (~3 months) and did not include baseline pre-infection kidney measures, limiting inference about onset versus pre-existing renal alterations [28].

Finally, kidney outcomes in children might be influenced by unique developmental and resilience factors: children have greater renal reserve, adaptive nephron hypertrophy potential, and perhaps different immunologic responses, which could mitigate progression compared to adults [11,12,23]. Nonetheless, evidence from adult cohorts shows that COVID-19–related AKI frequently leads to CKD or progressive eGFR decline [14,37,38], raising concern that subclinical injury in children may manifest later, particularly during growth or with additional renal insults.

Given the evolving but concerning signal, we recommend strengthening long-term renal surveillance and research methodology in paediatric populations. Prospective, long-term follow-up is needed, with cohorts undergoing serial measurements of eGFR, urine protein or albumin, blood pressure, and tubular injury markers over several years. Whenever feasible, studies should incorporate baseline pre-infection kidney function to help distinguish true post-infectious changes from pre-existing abnormalities. Future research should include stratified analyses by age group, pre-existing comorbidities such as chronic kidney disease, and severity of the acute illness, including AKI, the need for renal replacement therapy, or admission to intensive care.

From a clinical perspective, ongoing monitoring should be considered for children who experienced AKI or MIS-C during SARS-CoV-2 infection, even when apparent recovery is complete. Particular attention should be given to identifying subclinical renal injury, as routine urine screening for proteinuria or microhaematuria, assessment of tubular markers, and regular blood pressure evaluation may help detect early dysfunction before measurable declines in eGFR occur.

### 4.1. Strengths and Limitations

This systematic review has several important strengths. It is, to our knowledge, one of the few reviews focused specifically on long-term kidney outcomes in children aged 0–12 years following SARS-CoV-2 infection or MIS-C, an age group for which data are particularly scarce. The review followed PRISMA 2020 guidelines and a registered PROSPERO protocol, ensuring methodological transparency, pre-specified eligibility criteria, and a systematic search strategy across multiple databases. Rigorous screening and data extraction were performed independently by multiple reviewers, and risks of bias were assessed using established tools appropriate for observational study designs. In addition, this review synthesised findings across diverse clinical settings, including single-centre MIS-C cohorts, outpatient follow-up clinics, and large EHR-based populations, providing a broad overview of the current evidence landscape.

However, several limitations must be acknowledged. A key limitation is that several included MIS-C studies enrolled adolescents in addition to younger children, and long-term kidney outcomes were not consistently reported separately for the 0–12-year age group. The number of eligible studies was small, and most had modest sample sizes, limiting the precision and generalisability of the findings. Only one included study provided a contemporaneous uninfected control group, and none reported age-specific long-term kidney outcomes exclusively for the 0–12-year strata with sufficient granularity to allow meta-analysis. Heterogeneity in study design, follow-up duration, outcome definitions, and measurement methods further constrained the ability to synthesise data quantitatively. Many studies reported only crude laboratory values or single follow-up assessments without baseline measures or serial trajectories, preventing meaningful evaluation of trends in kidney function. In addition, most MIS-C studies lacked comprehensive assessment of proteinuria, tubular dysfunction, or blood pressure at long-term follow-up, and no study evaluated sustained eGFR decline or KDIGO-defined kidney disease progression in young children.

A further limitation is the short duration of follow-up in several studies, with only a minority assessing outcomes beyond six to twelve months; therefore, late-emerging renal sequelae cannot be excluded. The reliance on single-centre, retrospective cohorts also raises potential concerns regarding selection bias, incomplete follow-up, and variable ascertainment of kidney outcomes. Finally, large-scale EHR studies, while powerful, did not provide raw event counts or stratified kidney outcomes specifically for children aged 0–12 years, preventing definitive conclusions for this age group.

Taken together, these limitations indicate that the conclusions of this review should be interpreted as provisional rather than definitive, particularly with respect to long-term kidney outcomes in children aged 0–12 years. As larger, age-stratified cohorts with longer follow-up become available, the conclusions of this review may require re-evaluation or revision to reflect emerging evidence. Accordingly, these limitations highlight the need for robust, longitudinal, and age-stratified research to more fully characterise the long-term renal impact of SARS-CoV-2 infection in early childhood.

### 4.2. Clinical Implications

Although definitive conclusions are limited by the scarcity of age-specific long-term data, the available evidence suggests that structured renal follow-up may be most relevant for selected high-risk subgroups of children following SARS-CoV-2 infection. In particular, children who experienced severe acute COVID-19, MIS-C, or acute kidney injury during the acute phase may benefit from periodic blood pressure assessment and urinalysis during follow-up to detect potential subclinical renal abnormalities. These considerations are precautionary and should be interpreted in the context of current evidence limitations, pending further age-stratified prospective studies.

## 5. Conclusions

Large EHR-based studies in broader paediatric and adolescent populations (<21 years) suggest a modest but statistically significant increased risk of CKD following SARS-CoV-2 infection. In contrast, evidence specific to children aged 0–12 years remains limited to smaller MIS-C and mild COVID-19 cohorts, which generally demonstrate favourable short- to medium-term renal recovery but lack long-term, age-stratified outcome data. In summary, although early pandemic-era paediatric data suggested good renal recovery after SARS-CoV-2 infection or MIS-C, recent large-scale cohort data raise plausible concerns about subclinical kidney injury and an increased long-term risk of CKD in children. These findings caution against complacency and underscore the need for structured renal follow-up in paediatric COVID-19 survivors, especially those with AKI or MIS-C, to detect and mitigate potential chronic sequelae. However, because age-stratified data were limited and only a small subset of studies provided extractable outcomes for children aged 0–12 years, current evidence does not allow firm conclusions for this specific age group.

## Figures and Tables

**Table 1:** PICOS table for the search strategy in long-term kidney outcomes after SARS-CoV-2 infection in children aged 0–12 years.

Component	Description
Population (P)	Studies involving children aged 0–12 years with confirmed or probable SARS-CoV-2 infection, including acute COVID-19 or MIS-C/PIMS-TS. Studies with broader paediatric age ranges are eligible only if data for children ≤ 12 years are extractable or the median age is ≤12 years.
Intervention/Exposure (I)	Natural SARS-CoV-2 infection. This includes infection confirmed by PCR, antigen, serology, or clinical diagnosis. No therapeutic interventions are considered as exposures.
Comparison (C)	- Children without SARS-CoV-2 infection (matched or contemporaneous controls), OR - Pre-pandemic cohorts, OR - Pre/post comparison within the same child (baseline kidney function before infection vs. follow-up after infection).
Outcomes (O)	Primary outcomes: Incident chronic kidney disease (CKD) Decline in eGFR (continuous or categorical, e.g., ≥30% decline) Secondary outcomes: Persistent proteinuria or albuminuria Persistent haematuria New or persistent hypertension Kidney replacement therapy (dialysis) Composite adverse kidney outcomes Follow-up definitions: Post-acute: 30–89 days Early long-term: 90–179 days Long-term: ≥180 days (primary focus ≥90 days)
Study Design (S)	Observational studies with longitudinal follow-up, including prospective or retrospective cohort studies, case–control studies, and registry or electronic health record [EHR]-based cohort analyses. Excluded: randomised controlled trials, cross-sectional studies without follow-up, case reports, case series with <7 participants, qualitative studies, reviews, editorials, and non-human studies.

**Table 2:** Characteristics of included studies reporting long-term kidney outcomes after SARS-CoV-2 infection in children aged 0–12 years.

Author, Year	Country	Study Design	Sample Size (Total/Infected/Controls)	Age (Years) ^(b)^	COVID-19 Phenotype	Follow-Up Duration	Kidney Outcomes Assessed
**MIS-C/PIMS-TS cohorts**							
Lehman et al. [27], 2023	United States	Retrospective cohort	63/63/0	Mean 9.7 (SD 4.2)	MIS-C (CDC)	Median 8.5 months	Post-discharge hypertension; no long-term eGFR, CKD, proteinuria, or haematuria
Meneghel et al. [29], 2023	Italy	Retrospective MIS-C cohort	55/55/0	Mean 8 (range 1.2–17.5)	MIS-C (WHO)	6 months	Resolution of AKI and tubular dysfunction; no persistent proteinuria, haematuria, CKD, or KRT
Penner et al. [4], 2021	United Kingdom	Retrospective MIS-C/PIMS-TS cohort	46/46/0	Median 10.2 (IQR 8.8–13.3)	PIMS-TS (RCPCH)	6 weeks and 6 months	Creatinine, proteinuria, albumin, RBP/Cr; no CKD or eGFR trajectories reported
Zahir et al. [30], 2024	India	Retrospective MIS-C cohort	7/7/0	Median 11 (range 4–18)	MIS-C (CDC)	1 year	Complete renal recovery; no CKD, hypertension, proteinuria, haematuria, or KRT
Zuccotti et al. [31], 2023	Italy	Prospective MIS-C cohort	33/33/0	Median 10 (IQR 7–14)	MIS-C (CDC)	6 months	Acute creatinine elevation only; no long-term renal function, proteinuria, haematuria, or BP outcomes
**Acute COVID-19 cohorts**							
Li et al. [26], 2025 ^(a)^	United States	Multicentre EHR-based cohort	1,900,146/487,378/1,412,768	Mean 8.2 (SD 6.2)	Acute COVID-19	28–729 days	CKD incidence and eGFR decline (reported for <21 years; not extractable for 0–12 years)
Marcellino et al. [28], 2025	Italy	Cross-sectional post-COVID clinic	199/148/51	Mean 10.1 (SD 4.2)	Mild acute COVID-19	Median 3 months	Single assessment of measured GFR, proteinuria, microhaematuria; no longitudinal outcomes

^(a)^ Li et al. (2025) reported outcomes for the full <21-year cohort. Age-specific counts for children 0–12 years were not provided [26]. ^(b)^ Several MIS-C cohorts included adolescents up to 17–18 years of age; age-specific renal outcomes for children aged 0–12 years were not separately reported in these studies. Abbreviations: AKI, acute kidney injury; BP, blood pressure; CDC, Centers for Disease Control and Prevention; CKD, chronic kidney disease; COVID-19, coronavirus disease 2019; eGFR, estimated glomerular filtration rate; EHR, electronic health record; GFR, glomerular filtration rate; IQR, interquartile range; KRT, kidney replacement therapy; MIS-C, multisystem inflammatory syndrome in children; PIMS-TS, paediatric inflammatory multisystem syndrome temporally associated with SARS-CoV-2; RBP/Cr, retinol-binding protein-to-creatinine ratio; RCPCH, Royal College of Paediatrics and Child Health; SD, standard deviation; WHO, World Health Organization.

**Table 3:** Long-term kidney outcomes in children aged 0–12 years following SARS-CoV-2 infection.

Author, Year	Group	Follow-Up Window	N with Follow-Up	CKD Incidence	eGFR Change/≥30% Decline	Persistent Proteinuria	Persistent Haematuria	HTN	KRT Initiation	Composite Kidney Outcome	Notes
**MIS-C/PIMS-TS cohorts**											
Lehman et al. [27], 2023 ^(a)^	MIS-C	>30 days (median 8.5 mo)	50	Not reported	Not reported	Not reported	Not reported	14% (7/50)	0	Not reported	HTN was the only long-term renal outcome assessed
Meneghel et al. [29], 2023	MIS-C	6 months (≥180 d)	55	0	Not reported	0	0	1.8% (1/55)	0	Not defined	All renal abnormalities resolved except mild HTN in one child
Penner et al. [4], 2021	PIMS-TS	6 wk and 6 mo	6 wk: 46; 6 mo: 44	Not reported	Not reported	6 wk: 9%; 6 mo: 2%	Not reported	6 wk: 7%; 6 mo: 10%	0	Not reported	Creatinine normalised; no CKD or eGFR data
Zahir et al. [30], 2024	MIS-C with AKI	1 year (≥365 d)	7	0	Not reported	0	0	0	0	Not reported	Complete renal recovery in all children
Zuccotti et al. [31], 2023	MIS-C	6 months (≥180 d)	33	Not reported	Not reported	Not reported	Not reported	Not reported	0	Not reported	No persistent kidney abnormalities; no long-term renal testing performed
**Acute COVID-19 cohorts**											
Li et al. [26], 2025	Acute COVID-19	28–179 d and ≥180 d	Not extractable for 0–12 y	Not extractable	Not extractable	Not reported	Not reported	Not reported	Not reported	Not extractable	Only <21-year aggregate EHR outcomes available
Marcellino et al. [28], 2025	Mild COVID-19	Single assessment (median 3 mo)	148	Not assessed	Not applicable (no baseline)	52.7% (single measurement)	10.9% (single measurement)	Not reported	0	Not reported	Cross-sectional; chronicity cannot be determined

^(a)^ Kidney outcomes reflect the full MIS-C or PIMS-TS cohort; age-specific results for children 0–12 years were not reported and cannot be separated [27]. Abbreviations: AKI, acute kidney injury; CKD, chronic kidney disease; COVID-19, coronavirus disease 2019; d, days; eGFR, estimated glomerular filtration rate; EHR, electronic health record; HTN, hypertension; KRT, kidney replacement therapy; MIS-C, multisystem inflammatory syndrome in children; mo, month; PIMS-TS, paediatric inflammatory multisystem syndrome temporally associated with SARS-CoV-2; wk, week.

**Table 4:** Comparative effect estimates for long-term kidney outcomes after SARS-CoV-2 infection.

Author, Year	Outcome	Follow-Up Window	Comparison	Effect Measure	Effect Size (95% CI)	Adjusted?	Key Covariates Included	Notes
Lehman et al. [27], 2023	No comparative outcomes reported	>30 days (median 8.5 mo)	N/A	N/A	N/A	N/A	N/A	Single-arm MIS-C cohort; post-discharge HTN prevalence 14%
Li et al. [26], 2025 ^(a)^	New-onset CKD (stage ≥ 2)	28–729 d	COVID-19+ vs. COVID-19−	HR	1.17 (1.12–1.22)	Yes	Age, sex, race/ethnicity, PMCA category, obesity, healthcare utilisation, vaccination, comorbidities	Full <21-year cohort; no 0–12 y stratification
Li et al. [26], 2025 ^(a)^	New-onset CKD (stage ≥ 3)	28–729 d	COVID-19+ vs. COVID-19−	HR	1.35 (1.13–1.62)	Yes	Same as above	Same
Li et al. [26], 2025 ^(a)^	Composite kidney outcome * (pre-existing CKD)	28–179 d	COVID-19+ vs. COVID-19−	HR	1.15 (1.04–1.27)	Yes	Same as above	Applies to CKD subgroup (<21 y)
Li et al. [26], 2025 ^(a)^	Composite kidney outcome * (pre-existing CKD)	180–729 d	COVID-19+ vs. COVID-19−	HR	1.14 (1.06–1.22)	Yes	Same as above	Same
Li et al. [26], 2025 ^(a)^	eGFR decline ≥ 30%	28–179 d	COVID-19+ vs. COVID-19−	HR	1.14 (1.03–1.25)	Yes	Same as above	<21-year cohort; non-stratified
Li et al. [26], 2025 ^(a)^	eGFR decline ≥ 30%	180–729 d	COVID-19+ vs. COVID-19−	HR	1.13 (1.05–1.20)	Yes	Same as above	Same
Li et al. [26], 2025 ^(a)^	eGFR decline ≥ 40%	90–179 d	COVID-19+ vs. COVID-19−	HR	1.24 (1.10–1.41)	Yes	Same as above	AKI subgroup (<21 y)
Li et al. [26], 2025 ^(a)^	eGFR decline ≥ 40%	180–729 d	COVID-19+ vs. COVID-19−	HR	1.41 (1.21–1.63)	Yes	Same as above	Same
Li et al. [26], 2025 ^(a)^	eGFR decline ≥ 50%	90–179 d	COVID-19+ vs. COVID-19−	HR	1.47 (1.06–2.03)	Yes	Same as above	Same
Li et al. [26], 2025 ^(a)^	eGFR decline ≥ 50%	180–729 d	COVID-19+ vs. COVID-19−	HR	1.42 (1.31–1.55)	Yes	Same as above	Same
Li et al. [26], 2025 ^(a)^	Composite kidney outcome * (AKI subgroup)	90–179 d	COVID-19+ vs. COVID-19−	HR	1.29 (1.21–1.38)	Yes	Same as above	Applies only to acute-phase AKI subgroup
Li et al. [26], 2025 ^(a)^	Composite kidney outcome * (AKI subgroup)	180–729 d	COVID-19+ vs. COVID-19−	HR	1.33 (1.21–1.47)	Yes	Same as above	Same
Other included studies (Marcellino 2025 [28]; Meneghel 2023 [29]; Penner 2021 [4]; Zahir 2024 [30]; Zuccotti 2023 [31])	None reported	N/A	N/A	N/A	N/A	N/A	N/A	All were single-arm cohorts without comparator groups

* Composite kidney outcome included ≥50% eGFR decline, eGFR ≤ 15 mL/min/1.73 m^2^, dialysis, transplant, or development of ESKD. ^(a)^ Effect estimates derive from the overall <21-year cohort in Li et al. (2025); age-specific hazard ratios for children 0–12 years were not available [26]. Abbreviations: AKI, acute kidney injury; CKD, chronic kidney disease; CI, confidence interval; COVID-19, coronavirus disease 2019; d, days; eGFR, estimated glomerular filtration rate; HR, hazard ratio; HTN, hypertension; mo, month; N/A, not applicable; PMCA, Paediatric Medical Complexity Algorithm; SARS-CoV-2, severe acute respiratory syndrome coronavirus 2; y, years.

## Data Availability

All data generated or analysed during this study are included in this published article.

## Supplementary Materials

The following supporting information can be downloaded at: https://www.mdpi.com/article/10.3390/children13010075/s1, Table S1: Database-specific search syntax; Table S2: Definitions, diagnostic criteria, and kidney outcome measurement methods used in the included studies; Table S3: Newcastle–Ottawa Scale (NOS) quality assessment of included cohort and case–control studies; Table S4: ROBINS-I risk of bias assessment of included studies; Table S5: Articles that were excluded and the reason for exclusion; Table S6: Baseline characteristics of children aged 0–12 years in included studies reporting long-term kidney outcomes after SARS-CoV-2 infection; and Table S7: The PRISMA 2020 checklist.

## Abbreviations

The following abbreviations are used in this manuscript:

AAP, American Academy of Pediatrics; AKI, acute kidney injury; AKIN, Acute Kidney Injury Network; BP, blood pressure; CDC, Centers for Disease Control and Prevention; CKD, chronic kidney disease; COVID-19, coronavirus disease 2019; eGFR, estimated glomerular filtration rate; EHR, electronic health record; ESKD, end-stage kidney disease; FENa, fractional excretion of sodium; GFR, glomerular filtration rate; ICU, intensive care unit; IQR, interquartile range; IVIG, intravenous immunoglobulin; KDIGO, Kidney Disease: Improving Global Outcomes; KRT, kidney replacement therapy; LDH, lactate dehydrogenase; MIS-C, multisystem inflammatory syndrome in children; PASC, post-acute sequelae of SARS-CoV-2 infection; PCR, polymerase chain reaction; PICOS, Population, Intervention/Exposure, Comparison, Outcomes, Study design; PIMS-TS, paediatric inflammatory multisystem syndrome temporally associated with SARS-CoV-2; PrU/CrU, protein-to-creatinine ratio in urine; RBP, retinol-binding protein; RCPCH, Royal College of Paediatrics and Child Health; ROBINS-I, Risk OF Bias In Non-randomised Studies of Interventions; SCr, serum creatinine; SD, standard deviation; TD, tubular dysfunction; TRP, tubular reabsorption of phosphate; uNAG, urinary N-acetyl-β-D-glucosaminidase; WHO, World Health Organization.
