# Supplementary material for: Long-Term Kidney Outcomes After SARS-CoV-2 Infection in Children Aged 0–12 Years: A Systematic Review
Source: Children (Basel). 2026 Jan 2;13(1):75. doi: 10.3390/children13010075 (PMC12840186; doi:10.3390/children13010075)
Supplement: Supplementary file 1 [file children-13-00075-s001.zip › Supplementary Table S1.pdf]

Supplementary Table S1. Database-specific search syntax

Ovid MEDLINE(R) ALL <1946 to November 30, 2025>
1 exp COVID-19/ 305213<br>2 exp Coronavirus Infections/ 317841<br>3 exp Coronavirus/ 217975<br>4 exp SARS-CoV-2/ 202811<br>5 (covid-19 or covid19 or "covid 19" or sars-cov-2 or "sars cov 2" or coronavirus or "2019-ncov" or "severe acute respiratory syndrome coronavirus 2").ti,ab. 480056<br>6 1 or 2 or 3 or 4 or 5 502931<br>7 exp Child/ 2300106<br>8 exp Infant/ 1326633<br>9 exp Pediatrics/ 66101<br>10 (child* or infant* or toddler* or preschool* or paediatric* or pediatric* or "younger children" or "school-age").ti,ab. 2390620<br>11 7 or 8 or 9 or 103695312<br>12 (MIS-C or "MIS C" or PIMS-TS or "pediatric inflammatory multisystem syndrome" or "paediatric inflammatory multisystem syndrome" or "multisystem inflammatory syndrome").ti,ab. 3402<br>13 (MIS-C or "MIS C" or PIMS-TS).ti,ab. 2271<br>14 12 or 13 3402<br>15 exp Kidney Diseases/ 613243<br>16 exp Kidney Function Tests/ 91132<br>17 exp Glomerular Filtration Rate/ 54323<br>18 exp Proteinuria/ 44357<br>19 exp Hematuria/ 13109<br>20 exp Hypertension/ 335500<br>21 (kidney or renal or eGFR or "glomerular filtration rate" or creatinine or CKD or "chronic kidney" or proteinuria or albuminuria or hematuria or haematuria or hypertension).ti,ab. 1639914<br>22 15 or 16 or 17 or 18 or 19 or 20 or 21 1915188<br>23 ("long-term" or "long term" or longitudinal or "follow-up" or "post-acute" or "post acute").ti,ab. 2616689<br>24 6 and 11 and 22 and 23 254<br>25 6 and 11 and 14 and 23 514<br>26 24 or 25 729<br>27 limit 26 to (english language and humans and yr="2019 -Current") 510

Embase <1974 to 2025 November 30>
1 exp covid-19/ 492887<br>2 exp coronavirus infection/ 514568<br>3 exp sars related coronavirus/ 153581<br>4 exp sars cov 2/ 146237<br>5 (covid-19 or covid19 or "covid 19" or sars-cov-2 or "sars cov 2" or coronavirus or "2019-ncov" or "severe acute respiratory syndrome coronavirus 2").ti,ab. 560965<br>6 1 or 2 or 3 or 4 or 5 633884<br>7 exp child/ 3501779<br>8 exp infant/ 1262150<br>9 exp pediatrics/ 137133<br>10 (child* or infant* or toddler* or preschool* or paediatric* or pediatric* or "younger children" or "school age").ti,ab. 3119241<br>11 7 or 8 or 9 or 104424869

12 exp multisystem inflammatory syndrome/ 415067
13 (MIS-C or "MIS C" or PIMS-TS).ti,ab. 3431
14 12 or 13 415387
15 exp kidney disease/ 1406921
16 exp kidney function/ 290786
17 exp glomerular filtration rate/ 186658
18 exp proteinuria/ 149543
19 exp hematuria/ 71186
20 exp hypertension/ 1172007
21 (kidney or renal or eGFR or "glomerular filtration rate" or creatinine or CKD or "chronic kidney" or proteinuria or albuminuria or hematuria or haematuria or hypertension).ti,ab. 2464016
22 15 or 16 or 17 or 18 or 19 or 20 or 21 3452553
23 ("long-term" or "long term" or longitudinal or "follow-up" or "post-acute" or "post acute").ti,ab. 3960445
24 6 and 11 and 22 and 23 1138
25 6 and 11 and 14 and 23 1181
26 24 or 25 2094
27 limit 26 to (human and english language and yr="2019 -Current") 2042

CINHAL

S6 (((1 AND 2 AND 4 AND 5 OR (1 AND 2 AND 3 AND 5)) AND (S4 AND S5 AND S1 AND S2)) AND (S1 AND S2 AND S4 AND S5)) AND (S3 OR S5 OR S1 OR S2) (25)
S5 (TI ("long-term" OR "long term" OR longitudinal OR follow-up OR "post-acute" OR "post acute") OR AB ("long-term" OR "long term" OR longitudinal OR follow-up OR "post-acute" OR "post acute")) (637,216)
S4 (MH "Kidney Diseases" OR MH "Renal Insufficiency" OR MH "Kidney Function Tests" OR MH "Proteinuria" OR MH "Hematuria" OR MH "Hypertension-Pediatric" OR TI (kidney OR renal OR eGFR OR "glomerular filtration rate" OR creatinine OR CKD OR "chronic kidney" OR proteinuria OR albuminuria OR haematuria OR hematuria OR hypertension) OR AB (kidney OR renal OR eGFR OR "glomerular filtration rate" OR creatinine OR CKD OR "chronic kidney" OR proteinuria OR albuminuria OR haematuria OR hematuria OR hypertension)) (296,994)
S3 (MH "Multisystem Inflammatory Syndrome" OR TI (MIS-C OR "MIS C" OR PIMS-TS) OR AB (MIS-C OR "MIS C" OR PIMS-TS)) (837)
S2 (MH "Child" OR MH "Infant" OR MH "Pediatrics" OR TI (child* OR infant* OR toddler* OR preschool* OR paediatric* OR pediatric* OR "school-age" OR "younger children") OR AB (child* OR infant* OR toddler* OR preschool* OR paediatric* OR pediatric* OR "school-age" OR "younger children")) (1,060,044)
S1 (MH "COVID-19" OR MH "Coronavirus Infections" OR MH "Coronavirus" OR MH "SARS-CoV-2" OR TI (COVID-19 OR SARS-CoV-2 OR "SARS CoV 2" OR coronavirus OR "2019-nCoV") OR AB (COVID-19 OR SARS-CoV-2 OR "SARS CoV 2" OR coronavirus OR "2019-nCoV")) (169,021)

PubMed

7 Search: ((("COVID-19"[MeSH Terms] OR "sars cov 2"[MeSH Terms] OR "Coronavirus Infections"[MeSH Terms] OR "COVID-19"[Title/Abstract] OR "sars cov 2"[Title/Abstract] OR "sars cov 2"[Title/Abstract] OR "coronavirus"[Title/Abstract] OR "2019-nCoV"[Title/Abstract] OR "severe acute respiratory syndrome coronavirus 2"[Title/Abstract]) AND ("Child"[MeSH Terms] OR "Infant"[MeSH Terms] OR "Pediatrics"[MeSH Terms] OR "child*"[Title/Abstract] OR "infant*"[Title/Abstract] OR "toddler*"[Title/Abstract] OR "preschool*"[Title/Abstract] OR "paediatric*"[Title/Abstract] OR "pediatric*"[Title/Abstract] OR "school-age"[Title/Abstract] OR "younger children"[Title/Abstract]) AND ("mis c"[Title/Abstract] OR "mis c"[Title/Abstract] OR "PIMS-TS"[Title/Abstract] OR "paediatric inflammatory multisystem syndrome"[Title/Abstract] OR

"multisystem inflammatory syndrome in children"[Title/Abstract]) AND ("Kidney"[MeSH Terms] OR "Kidney Diseases"[MeSH Terms] OR "Renal Insufficiency"[MeSH Terms] OR "Acute Kidney Injury"[MeSH Terms] OR "Kidney"[Title/Abstract] OR "kidneys"[Title/Abstract] OR "renal"[Title/Abstract] OR "nephro*"[Title/Abstract] OR "chronic kidney disease"[Title/Abstract] OR "CKD"[Title/Abstract] OR "Acute Kidney Injury"[Title/Abstract] OR "AKI"[Title/Abstract] OR "eGFR"[Title/Abstract] OR "glomerular filtration rate"[Title/Abstract] OR "proteinuria"[Title/Abstract] OR "albuminuria"[Title/Abstract] OR "haematuria"[Title/Abstract] OR "hematuria"[Title/Abstract]) AND ("Follow-Up Studies"[MeSH Terms] OR "post-acute"[Title/Abstract] OR "post-acute"[Title/Abstract] OR "long-term"[Title/Abstract] OR "long-term"[Title/Abstract] OR "post covid"[Title/Abstract] OR "post covid"[Title/Abstract] OR "post-acute sequelae"[Title/Abstract] OR "PASC"[Title/Abstract] OR "persistent"[Title/Abstract] OR "outcome*"[Title/Abstract] OR "recovery"[Title/Abstract] OR "90 days"[Title/Abstract] OR "3-month"[Title/Abstract] OR "3-month"[Title/Abstract])) NOT ("animals"[MeSH Terms] NOT "humans"[MeSH Terms])) AND 2019/12/01:2025/12/31[Date - Publication] AND "english"[Language] (95)

6 Search: (("COVID-19"[MeSH Terms] OR "sars cov 2"[MeSH Terms] OR "Coronavirus Infections"[MeSH Terms] OR "COVID-19"[Title/Abstract] OR "sars cov 2"[Title/Abstract] OR "sars cov 2"[Title/Abstract] OR "coronavirus"[Title/Abstract] OR "2019-nCoV"[Title/Abstract] OR "severe acute respiratory syndrome coronavirus 2"[Title/Abstract]) AND ("Child"[MeSH Terms] OR "Infant"[MeSH Terms] OR "Pediatrics"[MeSH Terms] OR "child*"[Title/Abstract] OR "infant*"[Title/Abstract] OR "toddler*"[Title/Abstract] OR "preschool*"[Title/Abstract] OR "paediatric*"[Title/Abstract] OR "pediatric*"[Title/Abstract] OR "school-age"[Title/Abstract] OR "younger children"[Title/Abstract]) AND ("mis c"[Title/Abstract] OR "mis c"[Title/Abstract] OR "PIMS-TS"[Title/Abstract] OR "paediatric inflammatory multisystem syndrome"[Title/Abstract] OR "multisystem inflammatory syndrome in children"[Title/Abstract]) AND ("Kidney"[MeSH Terms] OR "Kidney Diseases"[MeSH Terms] OR "Renal Insufficiency"[MeSH Terms] OR "Acute Kidney Injury"[MeSH Terms] OR "Kidney"[Title/Abstract] OR "kidneys"[Title/Abstract] OR "renal"[Title/Abstract] OR "nephro*"[Title/Abstract] OR "chronic kidney disease"[Title/Abstract] OR "CKD"[Title/Abstract] OR "Acute Kidney Injury"[Title/Abstract] OR "AKI"[Title/Abstract] OR "eGFR"[Title/Abstract] OR "glomerular filtration rate"[Title/Abstract] OR "proteinuria"[Title/Abstract] OR "albuminuria"[Title/Abstract] OR "haematuria"[Title/Abstract] OR "hematuria"[Title/Abstract]) AND ("Follow-Up Studies"[MeSH Terms] OR "post-acute"[Title/Abstract] OR "post-acute"[Title/Abstract] OR "long-term"[Title/Abstract] OR "long-term"[Title/Abstract] OR "post covid"[Title/Abstract] OR "post covid"[Title/Abstract] OR "post-acute sequelae"[Title/Abstract] OR "PASC"[Title/Abstract] OR "persistent"[Title/Abstract] OR "outcome*"[Title/Abstract] OR "recovery"[Title/Abstract] OR "90 days"[Title/Abstract] OR "3-month"[Title/Abstract] OR "3-month"[Title/Abstract])) NOT ("animals"[MeSH Terms] NOT "humans"[MeSH Terms]) (96)

5 Search: ("COVID-19"[MeSH Terms] OR "sars cov 2"[MeSH Terms] OR "Coronavirus Infections"[MeSH Terms] OR "COVID-19"[Title/Abstract] OR "sars cov 2"[Title/Abstract] OR "sars cov 2"[Title/Abstract] OR "coronavirus"[Title/Abstract] OR "2019-nCoV"[Title/Abstract] OR "severe acute respiratory syndrome coronavirus 2"[Title/Abstract]) AND ("Child"[MeSH Terms] OR "Infant"[MeSH Terms] OR "Pediatrics"[MeSH Terms] OR "child*"[Title/Abstract] OR "infant*"[Title/Abstract] OR "toddler*"[Title/Abstract] OR "preschool*"[Title/Abstract] OR "paediatric*"[Title/Abstract] OR "pediatric*"[Title/Abstract] OR "school-age"[Title/Abstract] OR "younger children"[Title/Abstract]) AND ("mis c"[Title/Abstract] OR "mis c"[Title/Abstract] OR "PIMS-TS"[Title/Abstract] OR "paediatric inflammatory multisystem syndrome"[Title/Abstract] OR "multisystem inflammatory syndrome in children"[Title/Abstract]) AND ("Kidney"[MeSH Terms] OR "Kidney Diseases"[MeSH Terms] OR "Renal Insufficiency"[MeSH Terms] OR "Acute Kidney Injury"[MeSH Terms] OR "Kidney"[Title/Abstract] OR "kidneys"[Title/Abstract] OR "renal"[Title/Abstract] OR "nephro*"[Title/Abstract] OR "chronic kidney disease"[Title/Abstract]

OR "CKD"[Title/Abstract] OR "Acute Kidney Injury"[Title/Abstract] OR "AKI"[Title/Abstract] OR "eGFR"[Title/Abstract] OR "glomerular filtration rate"[Title/Abstract] OR "proteinuria"[Title/Abstract] OR "albuminuria"[Title/Abstract] OR "haematuria"[Title/Abstract] OR "hematuria"[Title/Abstract]) AND ("Follow-Up Studies"[MeSH Terms] OR "post-acute"[Title/Abstract] OR "post-acute"[Title/Abstract] OR "long-term"[Title/Abstract] OR "long-term"[Title/Abstract] OR "post covid"[Title/Abstract] OR "post covid"[Title/Abstract] OR "post-acute sequelae"[Title/Abstract] OR "PASC"[Title/Abstract] OR "persistent"[Title/Abstract] OR "outcome*"[Title/Abstract] OR "recovery"[Title/Abstract] OR "90 days"[Title/Abstract] OR "3-month"[Title/Abstract] OR "3-month"[Title/Abstract]) (96)

4 Search: ("COVID-19"[MeSH Terms] OR "sars cov 2"[MeSH Terms] OR "Coronavirus Infections"[MeSH Terms] OR "COVID-19"[Title/Abstract] OR "sars cov 2"[Title/Abstract] OR "sars cov 2"[Title/Abstract] OR "coronavirus"[Title/Abstract] OR "2019-nCoV"[Title/Abstract] OR "severe acute respiratory syndrome coronavirus 2"[Title/Abstract]) AND ("Child"[MeSH Terms] OR "Infant"[MeSH Terms] OR "Pediatrics"[MeSH Terms] OR "child*"[Title/Abstract] OR "infant*"[Title/Abstract] OR "toddler*"[Title/Abstract] OR "preschool*"[Title/Abstract] OR "paediatric*"[Title/Abstract] OR "pediatric*"[Title/Abstract] OR "school-age"[Title/Abstract] OR "younger children"[Title/Abstract]) AND ("mis c"[Title/Abstract] OR "mis c"[Title/Abstract] OR "PIMS-TS"[Title/Abstract] OR "paediatric inflammatory multisystem syndrome"[Title/Abstract] OR "multisystem inflammatory syndrome in children"[Title/Abstract]) AND ("Kidney"[MeSH Terms] OR "Kidney Diseases"[MeSH Terms] OR "Renal Insufficiency"[MeSH Terms] OR "Acute Kidney Injury"[MeSH Terms] OR "Kidney"[Title/Abstract] OR "kidneys"[Title/Abstract] OR "renal"[Title/Abstract] OR "nephro*"[Title/Abstract] OR "chronic kidney disease"[Title/Abstract] OR "CKD"[Title/Abstract] OR "Acute Kidney Injury"[Title/Abstract] OR "AKI"[Title/Abstract] OR "eGFR"[Title/Abstract] OR "glomerular filtration rate"[Title/Abstract] OR "proteinuria"[Title/Abstract] OR "albuminuria"[Title/Abstract] OR "haematuria"[Title/Abstract] OR "hematuria"[Title/Abstract]) (189)

3 Search: ("COVID-19"[MeSH Terms] OR "sars cov 2"[MeSH Terms] OR "Coronavirus Infections"[MeSH Terms] OR "COVID-19"[Title/Abstract] OR "sars cov 2"[Title/Abstract] OR "sars cov 2"[Title/Abstract] OR "coronavirus"[Title/Abstract] OR "2019-nCoV"[Title/Abstract] OR "severe acute respiratory syndrome coronavirus 2"[Title/Abstract]) AND ("Child"[MeSH Terms] OR "Infant"[MeSH Terms] OR "Pediatrics"[MeSH Terms] OR "child*"[Title/Abstract] OR "infant*"[Title/Abstract] OR "toddler*"[Title/Abstract] OR "preschool*"[Title/Abstract] OR "paediatric*"[Title/Abstract] OR "pediatric*"[Title/Abstract] OR "school-age"[Title/Abstract] OR "younger children"[Title/Abstract]) AND ("mis c"[Title/Abstract] OR "mis c"[Title/Abstract] OR "PIMS-TS"[Title/Abstract] OR "paediatric inflammatory multisystem syndrome"[Title/Abstract] OR "multisystem inflammatory syndrome in children"[Title/Abstract]) (2,750)

2 Search: ("COVID-19"[MeSH Terms] OR "sars cov 2"[MeSH Terms] OR "Coronavirus Infections"[MeSH Terms] OR "COVID-19"[Title/Abstract] OR "sars cov 2"[Title/Abstract] OR "sars cov 2"[Title/Abstract] OR "coronavirus"[Title/Abstract] OR "2019-nCoV"[Title/Abstract] OR "severe acute respiratory syndrome coronavirus 2"[Title/Abstract]) AND ("Child"[MeSH Terms] OR "Infant"[MeSH Terms] OR "Pediatrics"[MeSH Terms] OR "child*"[Title/Abstract] OR "infant*"[Title/Abstract] OR "toddler*"[Title/Abstract] OR "preschool*"[Title/Abstract] OR "paediatric*"[Title/Abstract] OR "pediatric*"[Title/Abstract] OR "school-age"[Title/Abstract] OR "younger children"[Title/Abstract]) (48,921)

1 Search: "COVID-19"[MeSH Terms] OR "sars cov 2"[MeSH Terms] OR "Coronavirus Infections"[MeSH Terms] OR "COVID-19"[Title/Abstract] OR "sars cov 2"[Title/Abstract] OR "sars cov 2"[Title/Abstract] OR "coronavirus"[Title/Abstract] OR "2019-nCoV"[Title/Abstract] OR "severe acute respiratory syndrome coronavirus 2"[Title/Abstract] (506,393)
