# Supplementary material for: Long-Term Kidney Outcomes After SARS-CoV-2 Infection in Children Aged 0–12 Years: A Systematic Review
Source: Children (Basel). 2026 Jan 2;13(1):75. doi: 10.3390/children13010075 (PMC12840186; doi:10.3390/children13010075)
Supplement: Supplementary file 1 [file children-13-00075-s001.zip › Supplementary Table S2.pdf]

Supplementary Table S2. Definitions, diagnostic criteria, and kidney outcome measurement methods used in the included studies.

Author, Year	SARS-CoV-2 case definition	MIS-C / PIMS-TS definition	eGFR equation used	CKD definition	AKI definition	Kidney outcomes measured	Methods of measurement	Follow-up timepoints
**MIS-C / PIMS-TS cohorts**								
Lehman et al. [26], 2023	PCR or antibody-confirmed SARS-CoV-2 exposure	CDC MIS-C criteria	Bedside Schwartz	Not assessed (CKD excluded)	KDIGO 2012 creatinine-based	SCr, eGFR, AKI, post-discharge hypertension	EHR labs; blood pressure using AAP 2017 percentiles	>30 days post-discharge (median 8.5 mo)
Meneghel et al. [29], 2023	PCR/serology or exposure, consistent with WHO	WHO MIS-C criteria	Not calculated (SCr only)	Not assessed (no baseline CKD)	KDIGO creatinine-based + urine output	AKI, tubular dysfunction, proteinuria, haematuria	Serum creatinine, urea, electrolytes; uNAG, TRP, 24-h proteinuria; urine output	Baseline, during admission, 6 mo
Penner et al. [4], 2021	PCR/IgG positivity or epidemiological link	RCPCH PIMS-TS criteria	Not calculated (SCr only)	Not assessed	Not reported	Creatinine, proteinuria, albumin, RBP/Cr, blood pressure	Standard labs; urinalysis; BP per paediatric percentiles; RBP/Cr ratio	6 wks and 6 mo
Zahir et al. [30], 2024	CDC MIS-C definition with confirmed recent SARS-CoV-2	CDC MIS-C criteria	Not calculated	Not assessed	AKIN stages 1–3	AKI severity, creatinine, proteinuria, haematuria	Serum creatinine; dipstick; microscopy; inflammatory markers	Minimum 1-year follow-up
Zuccotti et al. [31], 2023	PCR/serology/antigen or confirmed exposure	CDC MIS-C criteria	Not calculated	Not assessed	Creatinine-based severity scores (<50% or >50% rise)	Acute creatinine elevation; no long-term renal tests	Serum creatinine only (no urine/eGFR)	~6-month follow-up
**Acute COVID-19 cohorts**								
Li et al. [27], 2025	Positive PCR/antigen/serology or EHR diagnosis;	MIS-C excluded from	U25 age–sex equations	CKD stage ≥2 or ≥3 (eGFR <90	Creatinine rise ≥0.3 mg/dL or ≥50%	New-onset CKD, eGFR decline (≥30–50%),	EHR creatinine, dialysis/transplant	28–179 d and 180–729 d

	controls required negative test	kidney cohort		or <60 twice ≥90 d apart)		composite renal outcome	codes; algorithm-derived eGFR	
Marcellino et al. [28], 2025	PCR-confirmed; antigen tests confirmed by PCR	Not applicable (MIS-C excluded)	Not applicable; measured creatinine clearance	Not applicable	Not assessed	Measured GFR, proteinuria, microhaematuria, tubular markers	24-h urine CrCl; dipstick/microscopy; FENa, TRP; renal ultrasound	Single assessment ≥2 wks post-recovery (median 3 mo)

Abbreviations: AAP, American Academy of Pediatrics; AKI, acute kidney injury; AKIN, Acute Kidney Injury Network; BP, blood pressure; CDC, Centers for Disease Control and Prevention; CKD, chronic kidney disease; CrCl, creatinine clearance; eGFR, estimated glomerular filtration rate; EHR, electronic health record; FENa, fractional excretion of sodium; GFR, glomerular filtration rate; IgG, immunoglobulin G; MIS-C, multisystem inflammatory syndrome in children; PIMS-TS, paediatric inflammatory multisystem syndrome temporally associated with SARS-CoV-2; RBP/Cr, retinol-binding protein-to-creatinine ratio; RCPCH, Royal College of Paediatrics and Child Health; SCr, serum creatinine; TRP, tubular reabsorption of phosphate; uNAG, urinary N-acetyl-β-D-glucosaminidase; WHO, World Health Organization.
