# Supplementary material for: Long-Term Kidney Outcomes After SARS-CoV-2 Infection in Children Aged 0–12 Years: A Systematic Review
Source: Children (Basel). 2026 Jan 2;13(1):75. doi: 10.3390/children13010075 (PMC12840186; doi:10.3390/children13010075)
Supplement: Supplementary file 1 [file children-13-00075-s001.zip › Supplementary Table S3.pdf]

Supplementary Table S3. Newcastle–Ottawa Scale (NOS) quality assessment of included cohort and case–control studies.

Author, year	Selection (0–4)	Comparability (0–2)	Outcome (0–3)	Total (0–9)	Overall risk of bias	Notes
Lehman et al. [26], 2023	3	1	3	7	Moderate	Single-centre MIS-C cohort; no control group; objective BP measurement; adequate follow-up
Marcellino et al. [28], 2025	3	1	3	7	Moderate	Strict inclusion/exclusion; healthy controls; cross-sectional design limits comparability
Meneghel et al. [29], 2023	3	1	3	7	Moderate	Retrospective MIS-C cohort; complete 6-month follow-up; no comparator group
Penner et al. [4], 2021	3	1	3	7	Moderate	Single-centre PIMS-TS cohort; outcomes measured objectively; no control group
Zahir et al. [30], 2024	3	1	3	7	Moderate	Small MIS-C AKI cohort; full 1-year follow-up; no comparator group
Zuccotti et al. [31], 2023	3	1	3	7	Moderate	Prospective MIS-C cohort; complete follow-up; kidney outcomes limited to acute changes

Abbreviations: AKI, acute kidney injury; BP, blood pressure; MIS-C, multisystem inflammatory syndrome in children; NOS, Newcastle–Ottawa Scale; PIMS-TS, paediatric inflammatory multisystem syndrome temporally associated with SARS-CoV-2.
