# Supplementary material for: Long-Term Kidney Outcomes After SARS-CoV-2 Infection in Children Aged 0–12 Years: A Systematic Review
Source: Children (Basel). 2026 Jan 2;13(1):75. doi: 10.3390/children13010075 (PMC12840186; doi:10.3390/children13010075)
Supplement: Supplementary file 1 [file children-13-00075-s001.zip › Supplementary Table S4.pdf]

Supplementary Table S4. ROBINS-I risk of bias assessment of included studies.

Author, year	Confounding	Selection of participants	Classification of exposure	Deviations from intended exposure	Missing data	Measurement of outcomes	Selection of reported results	Overall ROBINS-I judgment	Notes
Li et al. [27], 2025	Moderate	Moderate	Low	Low	Moderate	Low	Moderate	Moderate risk of bias	Large multicentre EHR study; robust PS adjustment; residual confounding possible; missing creatinine follow-up data; age-specific (0–12 y) outcomes unavailable

Abbreviations: EHR, electronic health record; PS, propensity score; ROBINS-I, Risk of Bias in Non-randomized Studies of Interventions; y, years.
