# Supplementary material for: Long-Term Kidney Outcomes After SARS-CoV-2 Infection in Children Aged 0–12 Years: A Systematic Review
Source: Children (Basel). 2026 Jan 2;13(1):75. doi: 10.3390/children13010075 (PMC12840186; doi:10.3390/children13010075)
Supplement: Supplementary file 1 [file children-13-00075-s001.zip › Supplementary Table S5.pdf]

Supplementary Table S5. Articles that were excluded and the reason for exclusion.

Article excluded	Reason for exclusion
Short- and medium-term longitudinal outcomes of children diagnosed with multisystem inflammatory syndrome in children – report from a single centre in Pakistan (Abbas et al. 2024)	No long-term kidney outcomes reported; study focuses on cardiac and inflammatory outcomes, with renal involvement only described acutely during MIS-C admission.
Frequency and clinical characteristics of kidney involvement in children with suspected COVID-19 infection: a single-center cross-sectional study (Abdelkareem et al. 2025)	Acute in-hospital AKI and urine abnormalities only; no post-acute or long-term kidney follow-up ≥30 days after infection.
Clinical and epidemiological features and severity markers in children admitted with multisystem inflammatory syndrome in children (MIS-C) in a tertiary care center in the United Arab Emirates (AboAlEla et al. 2023)	MIS-C cohort with short-term severity markers; kidney involvement considered only as acute organ dysfunction, with no longitudinal kidney outcomes beyond the acute episode.
Kidney involvement in pediatric COVID-19 cases: a single-center experience (Ağbaş et al. 2022)	Cross-sectional evaluation of hematuria, proteinuria, and elevated creatinine at presentation; no longitudinal follow-up of kidney function ≥30 days after infection.
Multisystemic inflammatory syndrome in children (MIS-C) with COVID-19 and kidney involvement: poor outcomes in a case series (Alghamdi et al. 2025)	Case series of 6 MIS-C patients (<7 participants) describing acute AKI and ICU course only; no post-acute or long-term kidney follow-up.
Liver function recovery of COVID-19 patients after discharge: a follow-up study (An et al. 2021)	Adult cohort with liver outcomes only; not paediatric and no kidney outcomes, therefore outside the review's population and outcome scope.
Multisystem inflammatory syndrome in children: follow-up of a cohort from North India (Awasthi et al. 2022)	MIS-C follow-up study focused on clinical symptoms and cardiac outcomes; no kidney function, proteinuria, haematuria, or CKD outcomes reported.
COVID-19 in children with kidney disease: a report of 2 cases (Basalely et al. 2021)	Case report/very small case series (n=2) of children with pre-existing kidney disease; does not meet minimum sample size (≥7) and reports only acute course.
Acute kidney injury in critically ill children and young adults with suspected SARS-CoV-2 infection (Basu et al. 2022)	ICU cohort describing AKI within the first 14 days and 28-day outcomes; no post-acute or long-term kidney follow-up ≥30 days, so acute-only kidney outcomes.
Pediatric glomerulopathy after COVID-19 vaccination: a case series and review of the literature (Chuang et al. 2023)	Exposure is COVID-19 vaccination rather than natural SARS-CoV-2 infection; glomerulopathy post-vaccine, so wrong exposure for this review.
Acute kidney injury in pediatric acute SARS-CoV-2 infection and multisystem inflammatory syndrome in children (MIS-C): Is there a difference? (Grewal et al., 2021)	No long-term kidney outcomes reported; study focuses on acute AKI prevalence, risk factors, and in-hospital recovery only.
Severe acute respiratory syndrome coronavirus 2 infection in children with renal involvement: clinical characteristics (David et al., 2020)	No long-term kidney outcomes reported; renal abnormalities were assessed only during acute admission.
Acute kidney injury in children with COVID-19: a retrospective study (Kari et al., 2021)	No long-term kidney outcomes reported; AKI and urinary changes were evaluated during hospitalization only.
Acute kidney injury in COVID-19-associated multisystem inflammatory syndrome in children (MIS-C) (Lipton et al., 2021)	No long-term kidney outcomes reported; AKI was tracked only during the MIS-C admission with recovery by discharge.
Long-term outcomes and immune profiling in children with multisystem inflammatory syndrome (MIS-C) (Jaxybayeva et al., 2023)	No kidney outcomes reported; follow-up evaluates cardiac, immune, somatic, and neurological findings only.
Course of renal involvement in the short term in children with COVID-19 (Martin et al., 2021)	Follow-up limited to ~3 months and does not assess long-term kidney outcomes; mild urinary findings only; broad age range (1–18 years) without extractable ≤12-year subgroup.
Acute kidney injury in pediatric patients with COVID-19: clinical features and outcome (Mirzaee et al., 2023)	No long-term kidney outcomes reported; evaluates AKI incidence and in-hospital renal complications without post-acute follow-up.
The impact of COVID-19 on renal resistive index in kidney transplant recipients (Karaaslan et al., 2023)	Wrong population; adult kidney-transplant recipients, not pediatric; no natural SARS-CoV-2 infection kidney outcomes relevant to review.
Risk of neuropsychiatric and related conditions associated with SARS-CoV-2 infection (Lu et al., 2025)	No kidney outcomes reported; study focuses exclusively on neuropsychiatric sequelae.
Impact of SARS-CoV-2 infection on renal involvement in children (Fisher et al., 2020)	No long-term kidney outcomes reported; renal assessment limited to acute findings during the index hospitalization.

Renal manifestations of COVID-19 in children (Mittal et al., 2022)	Narrative review with no original data; does not report longitudinal post-acute kidney outcomes and therefore cannot contribute to incidence or follow-up data.
Renal involvement in COVID-19 among Iranian children (Mohkam et al., 2021)	Cross-sectional hospital-based cohort assessing acute AKI during admission only; no ≥90-day or post-discharge kidney follow-up.
Clinical characteristics and outcomes in MIS-C: a 12-month prospective study (Nitescu et al., 2025)	Follow-up focuses on cardiac and pulmonary sequelae; no kidney outcomes beyond transient proteinuria at admission; no long-term renal function assessment.
Pathology findings in pediatric patients with COVID-19 and kidney dysfunction (Nomura et al., 2022)	Small case-series of biopsied children with acute AKI and glomerular disease; no systematic follow-up of renal outcomes; study not designed to evaluate post-COVID long-term kidney trajectory.
Acute kidney injury in critically ill children with COVID-19 and MIS-C (Özen et al., 2023)	PICU study focused on predictors of acute AKI; renal outcomes measured only during hospitalization; no ≥90-day follow-up of kidney recovery or chronic impairment.
Twenty-four-month multidisciplinary follow-up of MIS-C (Piñera et al., 2025)	Long-term follow-up but no kidney outcomes reported; assessments were limited to cardiac, inflammatory, nutritional, and psychological sequelae.
Multisystem inflammatory syndrome in children and acute kidney injury: Italian PICU study (Ricci et al., 2022)	Evaluates development and resolution of AKI during PICU stay only; no post-discharge renal assessment; insufficient for long-term kidney outcomes review.
Multisystem inflammatory syndrome in children: clinical presentation and outcomes (Sezer et al., 2022)	Reports AKI only during admission and early follow-up; long-term outcomes described but kidney outcomes not assessed; follow-up focuses on cardiac, GI, pulmonary, and neuropsychiatric sequelae.
COVID-19 and renal involvement in children: a retrospective study (Sorkhi et al., 2022)	Describes acute kidney impairment during admission only; does not include structured post-acute or ≥90-day renal follow-up.
Renal dysfunction in hospitalised children with COVID-19 (Stewart et al., 2020)	Assesses frequency of AKI and abnormal creatinine at presentation; no longitudinal kidney follow-up and no post-acute outcomes.
COVID-19 disease among children and young adults enrolled in the NAPRTCS registry (Twichell et al., 2024)	No long-term kidney outcomes reported; study examines acute COVID-19 infections in children with pre-existing CKD, dialysis, and kidney transplants, with kidney involvement assessed only during the acute episode.
The Impact of COVID-19 Pandemic Lockdown on the Relationship between Pediatric MAFLD and Renal Function (Valentino et al., 2023)	Wrong population and exposure; study investigates renal–metabolic changes in children with congenital CAKUT/CKD related to lockdown lifestyle effects, not SARS-CoV-2 infection, and does not report post-COVID kidney outcomes.
COVID-19 in pediatric kidney transplantation: follow-up report of the IROC (Varnell Jr. et al., 2023)	No long-term kidney outcomes following natural infection; study focuses on kidney transplant recipients and acute allograft/respiratory outcomes after SARS-CoV-2, without ≥30-day renal follow-up or pediatric ≤12-year extractable kidney outcomes.
Incidence and predictors of development of new-onset hypertension post COVID-19 disease (Vyas et al., 2023)	Adult cohort (ages 30–74) evaluating post-COVID hypertension, not pediatric population and no kidney outcomes; therefore, outside the review's age criteria and outcome scope.
Clinical characteristics and outcomes of children with COVID-19 in PICUs during the Omicron wave in Taiwan (Wu et al., 2025)	Acute critical-care study of PICU admissions; no post-acute or long-term kidney outcomes reported, and renal parameters are not assessed beyond the initial hospitalization.
Post-COVID-19 Condition as a Mass Disabling Event: Unifying Pathophysiology and Clinical Phenotypes (Yameen et al., 2025)	Narrative/systematic review of Long COVID mechanisms and phenotypes; not an original clinical study, contains no extractable pediatric kidney outcome data.
Retrospective evaluation of acute kidney injury in paediatric COVID-19 patients (Yazılıtaş et al., 2024)	Reports acute AKI during COVID-19 presentation only; no follow-up kidney function outcomes ≥30–90 days, making it ineligible for a review focused on post-acute kidney outcomes in children.
COVID-19 in Children with Chronic Kidney Disease: Does it Differ Much? (Baltu et al., 2024)	Study includes children with pre-existing chronic kidney disease and reports only the acute course of SARS-CoV-2 infection (e.g., mild/asymptomatic cases, acute AKI incidence). No post-acute or long-term kidney outcomes are provided, and the population is not generalizable to previously healthy children ≤12 years.
COVID-19 in Children with Chronic Kidney Disease: Findings from the UK Renal Registry (Plumb et al., 2021)	Reports only acute SARS-CoV-2 infections in children with pre-existing CKD; provides no post-acute or long-term kidney outcomes. Population consists exclusively of children with advanced CKD and

	kidney replacement therapy, and therefore does not meet inclusion criteria for evaluating long-term renal outcomes after COVID-19 in the general ≤12-year pediatric population.
Clinical and subclinical acute kidney injury in children with mild-to-moderate COVID-19 (Saygili et al., 2022)	Assesses only acute and subclinical kidney injury during hospitalization for COVID-19; no post-acute or long-term (≥30-/≥90-day) kidney outcomes reported, so does not meet inclusion criteria for long-term renal consequences in children ≤ 12 years.
Acute Kidney Injury in Paediatric Inflammatory Multisystem Syndrome Temporally Associated with SARS-CoV-2 (PIMS-TS) Is Not Associated with Progression to Chronic Kidney Disease (Stewart et al., 2022)	Reports AKI and short-term recovery only in PIMS-TS patients, with follow-up limited to approximately 6 months. Does not provide extractable long-term kidney outcomes specific to children ≤12 years, and the cohort includes a broad <18-year age range without age-stratified renal data for ≤12-year children.
Anti-Factor H Antibody–Associated Hemolytic Uremic Syndrome Following SARS-CoV-2 Infection (Khandelwal et al., 2022)	Describes a rare complement-mediated atypical HUS phenotype triggered by SARS-CoV-2 in children with pre-existing anti-FH aHUS susceptibility. Kidney outcomes reflect acute thrombotic microangiopathy and short-term recovery, not long-term post-COVID renal outcomes in the general pediatric population. The cohort (ages 4–13 years) is disease-specific and not eligible for inclusion under the review's criteria.
Green Nephrology: Environmentally Sustainable Kidney Care in Pediatrics (Kingdom et al., 2025)	Review article focused on environmental sustainability in pediatric nephrology; does not report clinical data, kidney outcomes, or post-acute renal sequelae following SARS-CoV-2 infection. No extractable pediatric COVID-19 long-term kidney outcomes.
Long-term Kidney Outcomes After COVID-19: A Matched Cohort Study Using the OpenSAFELY Platform (Mahalingasivam et al., 2025)	Adult population study (ages 18+); does not include children and therefore provides no extractable long-term kidney outcomes for pediatric patients ≤12 years. Study design, cohort characteristics, and outcome measures are entirely outside the scope of the pediatric-focused review.
Pediatric Liver and Kidney Transplantation in the Era of COVID-19: A Follow-up Study from a Tertiary Referral Center in Iran (Shafiekhani et al., 2021)	Study population consists exclusively of pediatric solid organ transplant recipients (kidney and liver), a highly specific immunosuppressed group. Follow-up (3 months) evaluates post-transplant complications and COVID-19 screening, not long-term kidney outcomes after natural SARS-CoV-2 infection. No incident or post-acute renal outcomes related to COVID-19 are reported in children ≤12 years, and the study does not meet inclusion criteria for general pediatric kidney follow-up after infection.
Acute Kidney Injury Following Multisystem Inflammatory Syndrome Associated With SARS-CoV-2 Infection in Children: A Systematic Review and Meta-analysis (Tripathi et al., 2023)	Systematic review/meta-analysis rather than original patient-level data; does not provide extractable long-term post-acute kidney outcomes for children ≤12 years. As a secondary evidence synthesis focused on acute AKI during MIS-C, it does not meet inclusion criteria for primary studies evaluating longitudinal renal outcomes after natural SARS-CoV-2 infection.
COVID-19: Experiences of Lockdown and Support Needs in Children and Young Adults with Kidney Conditions (Tse et al., 2021)	Survey-based study assessing psychosocial experiences, information needs, shielding, and support during the COVID-19 pandemic in children and young adults with chronic kidney conditions. Does not report clinical kidney outcomes, renal function measures, AKI incidence, or any post-acute / long-term kidney outcomes following SARS-CoV-2 infection. Therefore, not eligible for inclusion in a systematic review focused on long-term renal sequelae after COVID-19 in children ≤12 years.
Short-Term Complications and Post-Acute Sequelae in Hospitalized Paediatric Patients with COVID-19 and Obesity (Valenzuela et al., 2023)	Evaluates short-term COVID-19 severity and obesity-related complications (ICU admission, oxygen use, NIMV, superinfections) and post-acute symptoms such as dyspnea and muscle weakness. Does not assess kidney function or report long-term renal outcomes, and AKI, although mentioned, is confined to acute hospitalization without follow-up. The 0–6-month outcomes focus on respiratory and neuromuscular symptoms only. Therefore, there are no extractable post-acute or long-term kidney outcomes relevant to children ≤12 years.

Pulmonary Sequelae of SARS-CoV-2 Infection and Factors Associated with Persistent Abnormal Lung Function at Six Months After Infection: Prospective Cohort Study (Yazji et al., 2022)	Adult prospective cohort (≥18 years) evaluating pulmonary function, exercise capacity, and radiologic lung recovery at 3 and 6 months after COVID-19. The study does not include children, does not report kidney outcomes, and provides no extractable post-acute or long-term renal data relevant to pediatric patients ≤12 years.
Long COVID Associated with SARS-CoV-2 Reinfection Among Children and Adolescents in the Omicron Era (Zhang et al., 2025)	Large retrospective EHR-based study evaluating post-acute sequelae (PASC) after first vs. second SARS-CoV-2 infections in individuals <21 years. Outcomes include PASC diagnosis and 24 symptom clusters, but no kidney function measures, AKI follow-up, eGFR data, CKD outcomes, or long-term renal trajectories are reported. Kidney outcomes are only counted as incident AKI diagnoses from ICD codes, with no renal function follow-up and no extractable data for children ≤12 years.
Cardiovascular Post-Acute Sequelae of SARS-CoV-2 in Children and Adolescents: Cohort Study Using Electronic Health Records (Zhang et al., 2025)	Large RECOVER-EHR study focused exclusively on cardiovascular post-acute sequelae 28 to 179 days after infection. Assesses hypertension, arrhythmias, myocarditis, heart failure, cardiomyopathy, thrombotic disorders, chest pain, syncope, and palpitations. No kidney outcomes were measured; there were no eGFR data, no AKI follow-up, no CKD progression, and no renal function trajectories. The study also includes a broad <21-year population with no extractable kidney data for children ≤12 years.
Chronic Granulomatous Disease: A Cohort of 173 Patients—10-Years Single Center Experience from Egypt (Abd Elaziz et al., 2023)	Describes clinical, genetic, infectious, and inflammatory manifestations of chronic granulomatous disease (CGD). The study population consists of children with primary immunodeficiency, not SARS-CoV-2 infection, and no kidney outcomes related to COVID-19 are reported. The article contains no post-acute or long-term renal follow-up and does not evaluate kidney involvement after SARS-CoV-2 infection in children ≤12 years.
Clinical Course, Imaging Features, and Outcomes of COVID-19 in Kidney Transplant Recipients (Abrishami et al., 2020)	Case series of 12 adult kidney transplant recipients with COVID-19, assessing symptoms, CT findings, and acute clinical outcomes. The study population is not pediatric, includes no children, and reports no kidney function outcomes related to SARS-CoV-2 infection beyond acute transplant graft parameters at admission. There is no post-acute or long-term renal follow-up, and the study does not provide extractable kidney outcome data for children ≤12 years.
Acute Kidney Injury in Pediatric Patients Hospitalized with Acute COVID-19 and MIS-C (Basalely et al., 2021)	Reports acute AKI during hospitalization for COVID-19 or MIS-C in patients ≤18 years; kidney outcomes are limited to in-hospital AKI incidence, staging, and short-term resolution. The study does not evaluate post-acute or long-term kidney outcomes (≥30–90 days), provides no follow-up renal function data, and does not include extractable long-term outcomes specific to children ≤12 years.
Impact of Pre-existing Comorbidities and Viral Variants on Post-acute Sequelae of COVID-19 ('Long COVID') in Dutch Primary Care: A Retrospective Cohort Study (Berends et al., 2025)	Adult and adolescent primary-care cohort (ages 0–90+) evaluating Long COVID symptoms (fatigue, respiratory complaints, cognitive issues) using GP consultation records. The study does not assess kidney function, AKI, eGFR, CKD progression, or any renal outcomes. Although "kidney disease" is included as a baseline comorbidity, no post-COVID renal outcomes are measured, and no extractable kidney data for children ≤12 years are provided.
Features of 20,133 Hospitalised UK Patients with COVID-19 Using the ISARIC WHO Clinical Characterisation Protocol (Docherty et al., 2020)	Large adult-focused national cohort study with a median age of 73 years; only 1.5% were <18 years and age-specific outcomes for children ≤12 years were not reported or extractable. Kidney outcomes were analysed only through acute AKI on admission (page 3–4 tables) with no post-acute or long-term renal follow-up (≥30–90 days).
Post-discharge Outcomes of Hospitalized Children Diagnosed with Acute SARS-CoV-2 or MIS-C (Fink et al., 2024)	Prospective multicentre survey assessing symptoms, neurocognitive complaints, functional status, HRQOL, and healthcare utilization after SARS-CoV-2 or MIS-C hospitalization. The study does not evaluate kidney outcomes; there are no eGFR values, no AKI follow-up, no CKD progression, and no renal function measures at any timepoint. Kidney involvement is absent from all reported outcomes (see symptom list on pp. 3–4; Table 2; healthcare utilization pp. 6–8;

	HRQOL and functioning pp. 11–17). Therefore, no extractable post-acute or long-term renal outcomes for children ≤12 years are available, and the study does not meet inclusion criteria.
Short-term and Medium-term Clinical Outcomes of Multisystem Inflammatory Syndrome in Children: A Prospective Observational Cohort Study (Glazyrina et al., 2024)	Evaluates only MIS-C–related inflammation, cardiac findings, symptoms, and recovery at 2 and 6 weeks post-discharge; no assessment of kidney outcomes beyond a brief report of acute-phase creatinine/urea abnormalities. Follow-up focuses on inflammatory markers and cardiac imaging, but does not include eGFR, AKI persistence, CKD outcomes, or any long-term renal function trajectory. Therefore, the study provides no extractable post-acute or long-term kidney outcomes for children ≤12 years and does not meet inclusion criteria.
Short-, mid-, and long-term complications after multisystem inflammatory syndrome in children over a 24-month follow-up period in Lima, Peru, 2020–2022 (Alvarado-Gamarra et al., 2023)	Although this study reports 24-month follow-up after MIS-C, no kidney-specific outcomes were measured at any timepoint. The follow-up assessed neurological, hematological, psychiatric, dermatological, respiratory, and cardiac sequelae, but did not include eGFR, creatinine, AKI persistence, CKD progression, proteinuria, haematuria, or any renal function markers. Therefore, no extractable post-acute or long-term kidney outcomes relevant to the review question are available.
Long-term health outcome and quality of life in children with multisystem inflammatory syndrome (D'Auria et al., 2024)	This study includes a 12-month multidisciplinary follow-up after MIS-C but does not report any kidney-specific outcomes. Although "renal function" is listed within the metabolic panel during acute and follow-up assessments, the study does not present creatinine values, eGFR, AKI persistence, CKD incidence, proteinuria, haematuria, or any longitudinal renal measures. The results tables (Tables 2–4) confirm no renal outcomes were analysed or reported. Therefore, no extractable post-acute or long-term kidney outcomes for children ≤12 years are available, and the study does not meet the inclusion criteria.
Acute Kidney Injury in Pediatric Inflammatory Multisystem Syndrome Temporally Associated With SARS-CoV-2 (Deep et al., 2020)	This study focuses exclusively on acute kidney injury (AKI) during PICU admission, reporting renal function only within the first 7 days of hospitalization. All renal outcomes, including creatinine, eGFR, AKI staging, CRRT use, and evolution of AKI, are acute-phase only, as shown in Supplementary Table 1 (baseline AKI and renal parameters) and throughout the results (AKI evolution limited to days 1–7). The study does not provide any post-discharge, ≥90-day, medium-term, or long-term kidney outcomes. Because our systematic review includes only studies reporting post-acute or long-term kidney outcomes (≥90 days), this study does not meet the inclusion criteria.
Acute kidney injury in children with moderate–severe COVID-19 and MIS-C: a tertiary referral center experience (Ozturk et al., 2024)	This study evaluates only acute kidney injury (AKI) during hospitalization in children with COVID-19 and MIS-C. All kidney data, including creatinine, BUN, eGFR, AKI staging, KRT use, hypertension, proteinuria, and haematuria, are limited to the admission period and inpatient course. No follow-up beyond discharge was performed. The study does not report post-acute, ≥90-day, medium-term, or long-term kidney outcomes, which are required for inclusion in our systematic review. Therefore, this article is excluded because it provides acute-phase kidney outcomes only and no longitudinal kidney follow-up.
Two-year follow-up on multisystem inflammatory syndrome in children (MIS-C) (Roge-Gurecka et al., 2025)	This study provides long-term follow-up (3, 6, 12, and 24 months) of children with MIS-C, but it does not report any kidney-specific outcomes. All reported outcomes focus on sleep quality (KSQ), fatigue (CFQ-11), functional capacity (6-MWT), orthostatic intolerance testing (OIT), and neuropsychological and physical well-being. There are no data on creatinine, eGFR, AKI persistence or recurrence, CKD, proteinuria, haematuria, hypertension follow-up, or any renal function markers at any timepoint (3–24 months). Because our systematic review includes only studies reporting post-acute or long-term kidney outcomes (≥90 days) for children ≤12 years, this study does not meet the inclusion criteria.

A retrospective analysis of acute kidney injury in children with post-COVID-19 multisystem inflammatory syndrome: insights into promising outcomes (El-Halaby et al., 2024)	This study reports only acute kidney injury (AKI) during hospitalization in MIS-C patients. All kidney outcomes, including AKI staging, creatinine values, urine findings, dialysis use, renal biopsy, and recovery, are confined to the acute admission period. The follow-up described in the paper is short-term (up to 6 months) and limited to confirming return to baseline kidney function after discharge. Crucially, the study does not present any post-acute (≥90 days) or long-term kidney outcomes, such as eGFR trends, CKD incidence, proteinuria or haematuria persistence, hypertension, or sustained renal impairment. The study's focus is on acute AKI epidemiology, severity, predictors, and in-hospital outcomes, not long-term renal sequelae. Therefore, it does not meet the inclusion criteria for our systematic review, which requires extractable long-term or post-acute kidney outcomes in children ≤12 years.
Age-Specific Clinical and Laboratory Features and Renal Involvement in Children with MIS-C: A Single Tertiary Centre Experience from Vojvodina (Milanović et al., 2025)	Although this study reports renal findings in MIS-C such as AKI incidence (6.3%), creatinine clearance values, sterile pyuria, and 24-hour proteinuria measurements, it evaluates kidney involvement only during the acute hospitalization period. The one-year follow-up described in the paper reports overall clinical recovery, with only one child showing mild persistent proteinuria, but no structured or quantitative long-term kidney outcomes are presented. There is no follow-up eGFR, no CKD staging, no assessment of persistent AKI, and no long-term renal function markers. The study also does not report any of the outcomes required for inclusion in our systematic review, including post-acute kidney outcomes assessed at or beyond 90 days, long-term eGFR or creatinine trends, CKD incidence or progression, persistent proteinuria or haematuria in a reportable format, or longitudinal renal function trajectories. Therefore, the study does not meet inclusion criteria, as its renal data are limited to the acute phase with no extractable post-acute or long-term kidney outcomes for children 12 years and younger.
Clinical presentation and short-term outcomes of multisystemic inflammatory syndrome in children in Lagos, Nigeria (Sokunbi et al., 2022)	Although this study reports acute kidney involvement in MIS-C, including AKI in 32.1%, oliguria, haematuria, proteinuria, and elevated creatinine, all kidney findings are restricted to the acute phase during hospitalization. Although the authors conducted follow-up at 2 weeks, 6 weeks, 3 months, and 6 months, the follow-up assessments focused only on cardiac outcomes such as coronary dilation, ventricular function, and ECG abnormalities. The study does not report any renal outcomes beyond the acute illness, including post-acute (≥90-day) kidney function results, follow-up creatinine or eGFR, evaluation of persistent AKI, CKD progression, renal sequelae, long-term proteinuria or haematuria, or any renal-specific follow-up metrics at 3 or 6 months. Although AKI is common in this cohort, there are no extractable long-term or ≥90-day kidney outcomes, which are required for inclusion in our systematic review. Therefore, this article is excluded.
Progress on diagnosis and treatment of multisystem inflammatory syndrome in children (Peng & Zhou, 2025)	Narrative mini-review with no original patient data. The article does not report kidney outcomes of any kind, including creatinine, eGFR, AKI follow-up, CKD progression, proteinuria or haematuria, or any post-acute or long-term renal outcomes. As a secondary review without extractable data and without a cohort of children ≤12 years, it does not meet inclusion criteria for primary studies reporting long-term kidney outcomes after SARS-CoV-2 infection or MIS-C.
Evaluation of clinical and laboratory findings in MIS-C patients associated with COVID-19 (Farshidgohar et al., 2024)	Cross-sectional MIS-C study reporting only acute kidney findings during hospitalization (proteinuria, glucosuria, pyuria). No post-acute or long-term kidney outcomes (≥90 days), no follow-up creatinine or eGFR, and no persistent renal outcomes. Does not meet inclusion criteria for primary studies evaluating long-term kidney outcomes in children ≤12 years.
