# Supplementary material for: Long-Term Kidney Outcomes After SARS-CoV-2 Infection in Children Aged 0–12 Years: A Systematic Review
Source: Children (Basel). 2026 Jan 2;13(1):75. doi: 10.3390/children13010075 (PMC12840186; doi:10.3390/children13010075)
Supplement: Supplementary file 1 [file children-13-00075-s001.zip › Supplementary Table S6.pdf]

Supplementary Table S6. Baseline characteristics of children aged 0–12 years in included studies reporting long-term kidney outcomes after SARS-CoV-2 infection.

Author, year	Group (0–12 y subset where available)	N	Age (years)	Female (%)	Pre-existing CKD	Baseline kidney findings	AKI during acute illness	AKI definition	COVID-19 phenotype	Follow-up duration (window)
**MIS-C / PIMS-TS cohorts**										
Lehman et al. [26], 2023	MIS-C	63	Mean 9.7 ± 4.2	41.3%	0%	eGFR 101 ± 44.5; no proteinuria/haematuria reported	23.8%	KDIGO	MIS-C (CDC)	Median 8.5 mo (≥90 d)
Meneghel et al. [29], 2023	MIS-C	55	Mean 8 (range 1.2–17.5)	38.2%	0%	Proteinuria 9/13; microhaematuria 22%; SCr median 43 μmol/L	23%	KDIGO	MIS-C (WHO)	6 mo (≥180 d)
Penner et al. [4], 2021	PIMS-TS (≤12 subset n=31)	31	Median 9.3 (IQR 7.8–10.2)	35%	0%	Creatinine elevation common but no KDIGO categorisation; baseline urine not reported	Not formally assessed	Not reported	PIMS-TS (RCPCH)	6 wk (30–89 d), 6 mo (≥180 d)
Zahir et al. [30], 2024	MIS-C with AKI	7	Median 11 (range 4–18)	57.1%	0%	Proteinuria 14.3%; haematuria 28.5%	100%	AKIN	MIS-C (CDC)	1 yr (≥365 d)
Zuccotti et al. [31], 2023	MIS-C	33	Median 10 (IQR 7–14)	24.2%	0%	Creatinine rise <50% in 6; >50% in 4; no baseline CKD	30.3%	Creatinine-based (no KDIGO)	MIS-C (CDC)	6 mo (≥180 d)
**Acute COVID-19 cohorts**										
Li et al. [27], 2025[(a)]	<21 cohort (0–12 not separable)	487,378 infected	Mean 8.8 ± 6.4	50%	1.3%	Not reported for 0–12 y	0.8% (acute-phase)	SCr-based KDIGO	Acute COVID-19	28–729 d (post-acute & long-term)
Marcellino et al. [28], 2025	Mild COVID-19	148	Mean 10.1 ± 4.2	39.2%	0%	No prior kidney disease; post-infection measured GFR median 132.3	Not assessed	Not applicable	Mild acute COVID-19	Median 3 mo (mixed 30–179 d)

[a]Baseline characteristics for Li et al. (2025) reflect the full <21-year cohort; age-specific data for children 0–12 years were not reported.

Abbreviations: AKI, acute kidney injury; AKIN, Acute Kidney Injury Network; CDC, Centers for Disease Control and Prevention; CKD, chronic kidney disease; COVID-19, coronavirus disease 2019; eGFR, estimated glomerular filtration rate; GFR, glomerular filtration rate; IQR, interquartile range; KDIGO, Kidney Disease:

Improving Global Outcomes; MIS-C, multisystem inflammatory syndrome in children; PIMS-TS, paediatric inflammatory multisystem syndrome temporally associated with SARS-CoV-2; RCPCH, Royal College of Paediatrics and Child Health; SCr, serum creatinine; WHO, World Health Organization.
